# Efficacy and safety of maternal RSV vaccination against infant lower respiratory tract infections: a systematic review and meta-analysis

**DOI:** 10.3389/fpubh.2026.1935421

**Published:** 2026-09-18

**Authors:** Gokce Naz Kucukbas Ozonder, Arzu Yavuz

**Affiliations:** 1 Department of Obstetrics and Gynecology, Perinatology Division, Kocaeli City Hospital, Kocaeli, Türkiye; 2 Department of Obstetrics and Gynecology, Kocaeli City Hospital, University of Health Sciences, Kocaeli, Türkiye

**Keywords:** infant, lower respiratory tract infection, maternal immunisation, preterm birth, respiratory syncytial virus, RSV vaccine, systematic review, vaccine effectiveness

## Abstract

**Background:**

Respiratory syncytial virus (RSV) is the leading cause of infant lower respiratory tract infection (LRTI) and hospitalisation worldwide. Maternal RSV prefusion F (RSVpreF) vaccination protects infants through transplacental antibody transfer. We aimed to synthesise the efficacy, real-world effectiveness, and safety of maternal RSV vaccination for preventing infant LRTI, integrating randomised and observational evidence.

**Methods:**

We searched PubMed, Embase, Scopus, and Cochrane CENTRAL (to June 2026) for randomised controlled trials (RCTs) and observational studies of maternal RSV vaccination reporting infant LRTI, hospitalisation, or maternal–infant safety. A single reviewer, with large language model-assisted verification, screened records, extracted data, and assessed risk of bias (RoB 2; Newcastle–Ottawa Scale). Randomised and observational evidence were pooled separately using random-effects models; overlapping populations were resolved based on study periods and named participating sites. Certainty was rated with GRADE (PROSPERO CRD420261400053).

**Results:**

Twenty-four studies (9 RCTs enrolling approximately 18,600 pregnant women, 15 observational) were included; all 15 observational studies evaluated the licensed Pfizer vaccine (Abrysvo). Pooled vaccine effectiveness against infant RSV-associated hospitalisation was 79.7% (95% CI 73.9–84.2) within 3 months and 68.1% (57.1–76.3) through 6 months. Randomised efficacy against severe RSV-LRTI was 70% for the licensed Pfizer vaccine, closely concordant with the real-world effectiveness; efficacy against medically attended RSV-LRTI was 51.5% (36.3–63.1). Preterm birth was modestly increased in the randomised trials (Mantel–Haenszel RR 1.17, 95% CI 1.03–1.32), but the signal was not robust: it became non-significant after excluding the GSK trial (GRACE; 1.10, 0.95–1.27), and for the licensed Pfizer vaccine, the increase was small and not robustly significant (pivotal MATISSE trial 1.20, 0.98–1.46; three-trial Pfizer pool 1.21, 1.00–1.46), while observational data showed a biologically implausible protective association (0.90, 0.82–0.99), consistent with healthy vaccinee confounding. No other maternal or neonatal safety signal was identified. Certainty of evidence was moderate for effectiveness and efficacy.

**Conclusion:**

Maternal RSVpreF vaccination confers substantial, generalisable protection against infant RSV disease, with randomised and real-world evidence in close agreement. For the licensed Pfizer vaccine administered within its approved 32–36-week window, no robustly significant increase in preterm birth is established, supporting its continued programmatic use alongside ongoing post-marketing surveillance.

**Systematic review registration:**

Unique Identifier: CRD420261400053 https://www.crd.york.ac.uk/PROSPERO/view/CRD420261400053

## Introduction

Respiratory syncytial virus (RSV) is the leading cause of acute lower respiratory tract infection and of infection-related hospitalisation in infants worldwide, with the greatest burden of severe disease concentrated in the first months of life (1). Most infants hospitalised with RSV were previously healthy and born at term, and effective prevention in early infancy has long been a public health priority.

Because young infants respond poorly to direct vaccination, maternal immunisation during pregnancy, which protects the newborn through transplacental transfer of antibodies, is now a leading preventive strategy. A bivalent RSV prefusion F protein-based vaccine (RSVpreF; Pfizer) was licensed in 2023 for administration during pregnancy following the pivotal MATISSE trial (2). The development of other maternal candidates has been less straightforward: a GSK prefusion F vaccine (RSVPreF3-Mat) was not advanced for maternal use after an imbalance in preterm birth led to early trial termination (3), and an earlier Novavax RSV F nanoparticle vaccine did not meet its primary efficacy endpoint (4). Reflecting the preterm-birth signal observed across the trial programme, the licensed Pfizer vaccine is recommended only within a restricted 32–36-week gestational window (5).

At licensure, the evidence base consisted almost entirely of randomised controlled trials. A 2024 Cochrane review of maternal RSV vaccination, which was limited to randomised trials, concluded that the evidence would be substantially improved by well-designed observational studies with long-term safety follow-up (6). Since then, substantial real-world evidence has accumulated from national vaccination programmes in multiple countries, including case–control studies of effectiveness (predominantly test-negative) against infant RSV hospitalisation and large post-marketing cohorts assessing maternal and neonatal safety, which has not previously been synthesised alongside the randomised evidence.

We conducted this systematic review and meta-analysis to summarise the efficacy, real-world effectiveness, and safety of maternal RSV vaccination for the prevention of infant lower respiratory tract infection. We integrated randomised and observational evidence, analysed separately, to quantify effectiveness against infant RSV hospitalisation and efficacy against RSV lower respiratory tract illness, to evaluate maternal and neonatal safety (in particular preterm birth and hypertensive disorders of pregnancy), and to rate the certainty of the resulting evidence.

## Materials and methods

### Protocol and registration

This systematic review was conducted in accordance with the Preferred Reporting Items for Systematic Reviews and Meta-Analyses (PRISMA 2020) guidelines, and the completed PRISMA 2020 checklist is provided in Supplementary material S2. The protocol was registered prospectively with PROSPERO (registration number CRD420261400053). The registered protocol specified that screening, data extraction, and risk-of-bias assessment would each be carried out “independently by at least two people (or person–machine combination) with a process to resolve differences.” Because the second reviewer became unavailable, these steps were performed by a single human reviewer working with a large language model tool, the person–machine combination anticipated in the registration, as detailed below.

### Eligibility criteria

Studies were eligible if they enrolled pregnant women who received any respiratory syncytial virus (RSV) vaccine during pregnancy and their infants (Population and Intervention), and compared maternal RSV vaccination with placebo, no vaccination, or standard care (Comparator). Eligible studies reported at least one outcome of interest: the primary outcome was RSV-associated lower respiratory tract infection (LRTI) in infants; secondary outcomes included RSV-related hospitalisation, severe RSV infection, intensive care unit admission, infant mortality, maternal adverse events, pregnancy outcomes, and neonatal adverse events. Randomised controlled trials (RCTs), non-randomised comparative studies, and cohort studies were included. Animal studies, case reports and case series, reviews, editorials, conference abstracts without sufficient data, and non-English publications were excluded. Ecological before-after (impact) studies and descriptive burden or protection-gap studies were also excluded, as they do not estimate an individual-level vaccine effect. In every study reporting an RSV outcome (the effectiveness studies and the randomised trials), the RSV-specific outcomes were laboratory-confirmed from a respiratory specimen, predominantly by reverse transcriptase PCR, with antigen or immunofluorescence testing accepted in some effectiveness studies, rather than by clinical diagnosis alone.

### Information sources

Four electronic databases were searched from inception to 15 June 2026: PubMed/MEDLINE, Embase, the Cochrane Central Register of Controlled Trials (CENTRAL), and Scopus. The reference lists of included studies and relevant systematic reviews were screened manually to identify additional eligible records, and trial registry records retrieved through CENTRAL were reviewed to identify completed and ongoing studies.

### Search strategy

The search strategy was developed around three concepts combined with the Boolean operator AND: (i) respiratory syncytial virus, (ii) pregnancy or maternal status, and (iii) vaccination or immunisation. Within each concept, controlled vocabulary terms (Medical Subject Headings in PubMed/MEDLINE; Emtree terms in Embase) and free-text title/abstract/keyword terms were combined with OR. No date or study-design restrictions were applied. The complete, line-by-line search strategy for each database is provided in Supplementary material S1.

### Study selection and de-duplication

Records from all databases were combined and duplicates were removed using an automated procedure that matched records first on Digital Object Identifier (DOI) and, where a DOI was unavailable, on normalised title and publication year; any residual duplicates were removed during screening. A total of 5,415 records were identified, of which 2,556 were duplicates and 118 were retrievable only as non-machine-readable PDFs, leaving 2,741 unique records for screening. Titles and abstracts were screened against the eligibility criteria, and the full texts of potentially eligible records were then assessed. Screening was performed by a single reviewer; a large language model tool was used to assist with relevance prioritisation and as an independent verification check, with all final inclusion and exclusion decisions made by the reviewer. Reasons for exclusion at full-text assessment were recorded. The study selection process is summarised in a PRISMA 2020 flow diagram (Figure 1). Exclusion reasons were assigned hierarchically; where a report met more than one criterion, the first applicable reason was recorded.

**Figure 1:**
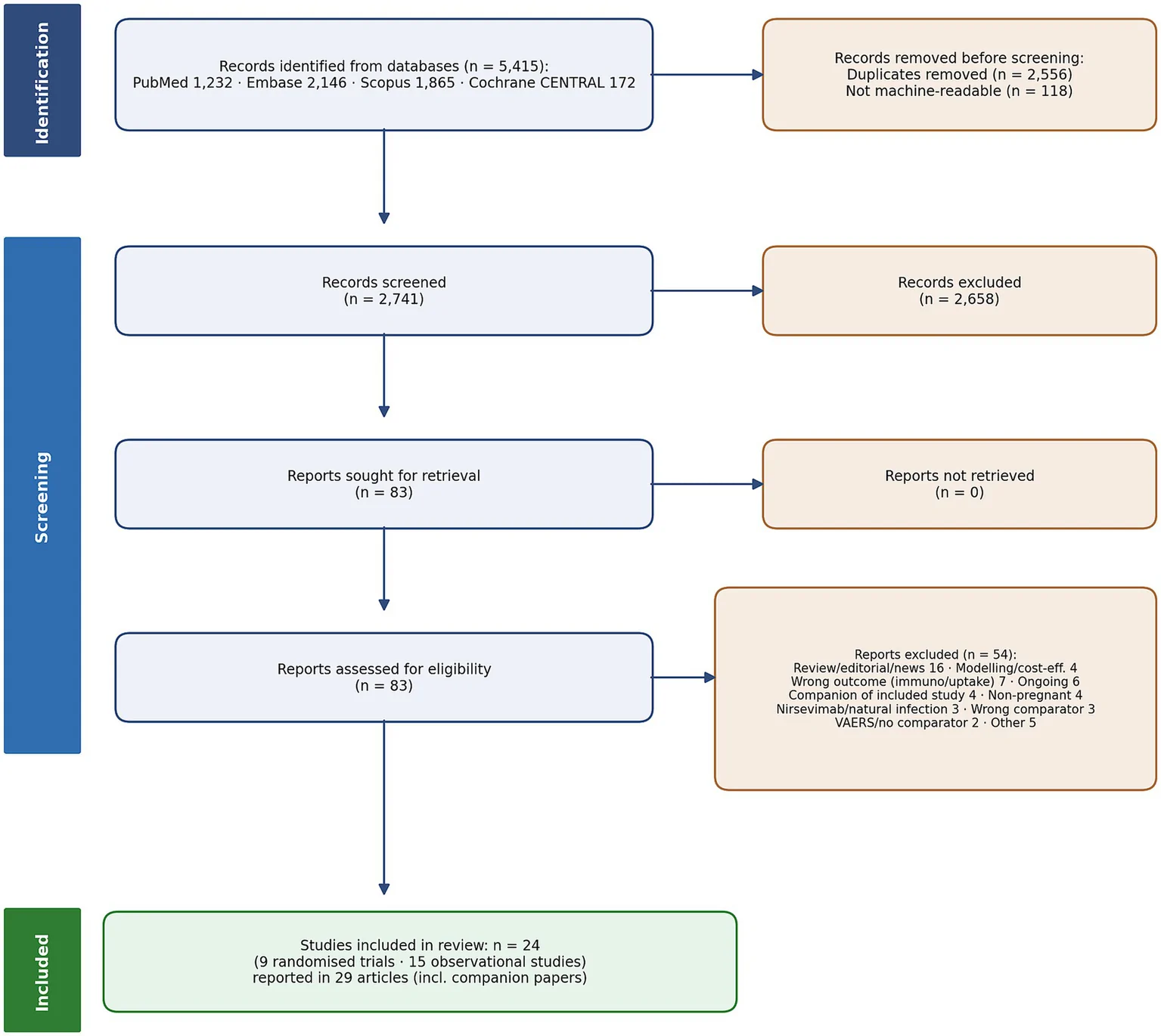
PRISMA 2020 flow diagram of study identification, screening, and inclusion.

### Data extraction

Data were extracted into a standardised, piloted extraction form. Extracted items included the first author, publication year, country, study design and phase, participant characteristics, vaccine type and timing of administration, comparator, outcome definitions and measures, duration of follow-up, and safety outcomes. Where multiple reports described the same trial population, data were extracted once to avoid double-counting, with the most complete report used as the primary source and companion reports used to supplement specific outcomes. Data extraction was performed by a single reviewer with large language model-assisted verification. Every quantitative value entering a pooled estimate was subsequently re-verified by the reviewer against the corresponding table or figure in the primary published source, and each check was documented in an audit worksheet. Where a study had initially been identified as a preprint or conference abstract, the peer-reviewed version of record was obtained and used for the final analysis. Discrepancies identified during this verification were corrected before pooling, and count-based randomised outcomes were re-pooled from the source event counts.

### Risk-of-bias assessment

Risk of bias was assessed at the outcome level. Randomised controlled trials were appraised using the Cochrane Risk of Bias 2 (RoB 2) tool, while non-randomised and cohort studies were appraised using the Newcastle–Ottawa Scale. Assessments were performed by a single reviewer.

### Effect measures and synthesis

For dichotomous outcomes, effect estimates were expressed as risk ratios (RR) or odds ratios (OR) with 95% confidence intervals; for continuous outcomes, they were expressed as mean differences or standardised mean differences with 95% confidence intervals. Where studies were sufficiently clinically and methodologically homogeneous, results were pooled using a DerSimonian–Laird random-effects model. Ratio measures were pooled on the natural logarithm scale by generic inverse-variance weighting and back-transformed for reporting. For outcomes pooled directly from trial event counts (such as preterm birth and other binary safety outcomes in the randomised trials), the Mantel–Haenszel method was used, which is preferable for sparse or unbalanced event data and accommodates zero-event arms without continuity correction, alongside the Cochran–Mantel–Haenszel test for the pooled effect. For test-negative and case–control studies, vaccine effectiveness was derived from the adjusted odds ratio as (1 − OR) × 100. Statistical heterogeneity was assessed using the I-squared statistic and the chi-squared-based Cochran’s Q test; because few studies contributed to each pool, these heterogeneity tests had limited statistical power and were interpreted alongside the I-squared value and inspection of the forest plots. The adjusted odds ratios, risk ratios, incidence rate ratios, and adjusted risk reductions reported by the observational studies were treated as approximately equivalent ratio measures for pooling; this approximation is reasonable given the generally low event rates observed, although odds ratios and risk ratios diverge somewhat for higher-incidence outcomes such as hypertensive disorders of pregnancy. Randomised and non-randomised studies were analysed separately and not pooled together. Where quantitative pooling was not appropriate, findings were synthesised narratively. Only pre-specified efficacy endpoints from trials designed to assess efficacy were eligible for the quantitative efficacy synthesis. Efficacy estimates that were exploratory or post-hoc and not pre-specified endpoints—for example, the exploratory efficacy results reported by the proof-of-concept phase 2b trial Simões et al. (7), which involved no formal statistical hypothesis testing—were not pooled and are reported narratively only. This rule was applied consistently across all included studies and was based on the status of each endpoint rather than the value of its result, to avoid selective outcome inclusion at the review level. Efficacy endpoints that were pre-specified but analysed on truncated data following early trial termination (e.g., GRACE/Dieussaert 2024) remained eligible for synthesis, with the resulting imprecision addressed in the certainty-of-evidence assessment.

### Subgroup, sensitivity, and publication bias analyses

Where data permitted, subgroup analyses were planned by type of RSV vaccine, gestational age at vaccination, preterm versus term infants, geographic region, and study design. Sensitivity analyses were planned by excluding studies at high risk of bias and by restricting analyses to randomised controlled trials. When at least 10 studies contributed to an outcome, publication bias was to be assessed by visual inspection of funnel plots and Egger’s test. The subgroup analysis of hypertensive disorders of pregnancy by degree of confounding adjustment was not pre-specified and is reported as an exploratory, post-hoc analysis.

### Certainty of evidence

The certainty of evidence for each key outcome was rated using the Grading of Recommendations Assessment, Development and Evaluation (GRADE) approach, considering risk of bias, inconsistency, indirectness, imprecision, and publication bias, and summarised in a Summary of Findings table.

### Deviations from the registered protocol

The registered protocol (PROSPERO CRD420261400053) specified that screening, data extraction, and risk-of-bias assessment would each be performed “independently by at least two people (or person–machine combination) with a process to resolve differences.” Because the second reviewer became unavailable due to illness, these steps were undertaken by a single human reviewer supported by a large language model tool, the person–machine combination anticipated in the registration, rather than by two independent human reviewers. To safeguard reliability, the language model served as an additional verification check, excluded records were re-screened after an interval, and all final inclusion, extraction, and appraisal decisions were made by, and remain the responsibility of, the human reviewer. This approach is therefore consistent with the registered protocol, and no protocol amendment was required. The only pre-specified analysis not carried out was formal publication bias testing (Egger’s test), which the protocol reserved for syntheses of at least ten studies—a threshold no single outcome reached. Two further minor departures from the registered protocol are noted for transparency. First, eligibility was extended from the registered publication cut-off of 30 May 2026 to include eligible studies published up to the search date of 15 June 2026, in order to capture the most recent evidence. Second, the meta-analyses were performed in Python rather than the registered RevMan/Stata software; this does not affect the validity of the random-effects estimates.

### Use of artificial intelligence

A large language model (Claude; developed by Anthropic, San Francisco, CA, USA; model Claude Opus 4.8) was used to support the conduct of this review. Specifically, the model assisted with the removal of duplicate records (matching on Digital Object Identifier and, where unavailable, on title and publication year), prioritisation of records during title and abstract screening, verification of screening and data extraction decisions against the source documents, organisation of data, statistical computation of the meta-analyses, and drafting and language editing of the manuscript text. The model did not make final eligibility, inclusion, exclusion, data extraction, or risk-of-bias judgements, and did not generate the scientific conclusions; all such decisions were made and verified against the primary sources by the human author, who reviewed and edited all AI-assisted outputs and takes full responsibility for the integrity and accuracy of the work.

### Handling of overlapping (non-independent) populations

Meta-analysis assumes each included study contributes independent data. Several studies shared patient populations (same databases, hospital networks, or region and season) and therefore could not all be included in the same pooled estimate without double-counting. For each overlapping cluster, we selected a single primary study for the main analysis, chosen on the basis of the largest/most complete population, the highest risk-of-bias rating, and the broadest catchment area, while retaining the remaining studies for sensitivity analyses only. Overlap was determined from reported study periods, geographic coverage, and, where available, the named participating sites rather than being assumed. The overlap assessment and resolution for each cluster are detailed in Supplementary material S7.

*United Kingdom effectiveness*. McLachlan (Scotland; whole-population national linkage, *n* = 27,565; admissions 12 Aug 2024–31 Mar 2025; Newcastle–Ottawa 9/9) and O’Hagan/UK2 (37 UK sites; admissions 2 Sep 2025–31 Jan 2026; season 2) cover different RSV seasons and are mutually independent; both were included in the primary analysis. Williams/UK1 (30 UK sites, including Scottish sites; admissions 30 Sep 2024–20 Jan 2025; season 1) overlaps with McLachlan in the 2024–25 season and was therefore reserved for sensitivity analysis, with McLachlan preferred (national coverage, larger sample, higher quality).

*Argentina effectiveness*. BERNI (Perez Marc; 12 hospitals across 7 provinces, *n* = 505; 2024 season; Newcastle–Ottawa 9/9), Razzini (RIMA network, 3 Buenos Aires-region hospitals; 2024), and Gentile (4 referral hospitals; 2024) all overlap. Inspection of the named sites confirmed shared hospitals across every pair (BERNI-Razzini: Elizalde and Ludovica; BERNI-Gentile: Vilela, Rosario; Razzini-Gentile: Alejandro Posadas), within the same 2024 season. BERNI was selected as the single primary study (national coverage encompassing the others, largest sample, highest quality with inverse-probability-of-treatment weighting); Razzini and Gentile were reserved for sensitivity analysis.

*Other overlaps*. The two TriNetX analyses (Kitano and Hsieh) draw on the same database; Kitano (full text, propensity score matched) was retained, and Hsieh was excluded from pooling. The remaining safety cohorts (Son, Sentinel, Utah, France, and Kitano/TriNetX) use distinct data sources and were treated as independent. The Vaccine Safety Datalink report (8) (conference abstract) was used for sensitivity/narrative synthesis only. Randomised trials were pooled separately from observational studies throughout. Data sources for the pooled safety cohorts were verified as distinct (Son: a single New York City health system; Utah: statewide vital records; France: the SNDS national database; Kitano: TriNetX). Utah’s statewide vital records cohort (9) is likewise independent. The Sentinel Common Data Model draws on five health plans, including Kaiser Permanente Northwest and HealthPartners, which are also member sites of the Vaccine Safety Datalink (VSD); thus, Sentinel and VSD share populations. As VSD is used for narrative/sensitivity purposes only, this does not affect the primary safety pool, but VSD was not combined with Sentinel in any quantitative analysis.

## Results

### Study selection (PRISMA flow)

The database searches identified 5,415 records (PubMed 1,232; Embase 2,146; Scopus 1,865; Cochrane CENTRAL 172). After the removal of 2,556 duplicates and 118 records that were not machine-readable, 2,741 records were screened by title and abstract. Eighty-three full-text reports were assessed for eligibility, of which 54 were excluded (most commonly reviews, editorials, or news items [*n* = 16], wrong outcome [*n* = 7], and ongoing studies without results [*n* = 6]); the full list of excluded full-text reports with reasons is provided in Supplementary material S4. The remaining 29 reports corresponded to 24 studies (9 randomised controlled trials and 15 observational studies; several trials contributed more than one companion report), which were included in the review (Figure 1); their characteristics are tabulated in Supplementary material S3. The nine randomised trials (reported across 14 articles) enrolled approximately 18,600 pregnant women and 18,300 liveborn infants; the three phase 3 efficacy trials, MATISSE (Pfizer), GRACE (GSK), and PREPARE (Novavax), accounted for 17,384 women and 17,119 infants. Because the observational studies used heterogeneous designs (case–control—test-negative and population-based—and cohort) and included partially overlapping populations, their participant numbers are reported per study in Table 1 and per analysis in the corresponding forest plots, rather than as a single pooled total, to ensure that no individual is counted more than once. Whereas the randomised trials evaluated three different vaccines (the Pfizer bivalent RSVpreF, the GSK RSVPreF3-Mat, and the Novavax RSV F nanoparticle vaccines), all 15 observational studies evaluated the licensed Pfizer bivalent RSVpreF vaccine (Abrysvo), the only maternal RSV vaccine approved for use in pregnancy, so the entire body of real-world effectiveness and safety evidence pertains specifically to the vaccine in clinical use. No additional eligible records were identified through reference-list screening or trial-registry review beyond those retrieved by the database searches.

**Table 1:** Characteristics of the 24 included studies (9 randomised controlled trials and 15 observational studies).

Study (first author, year)	Design	Country/setting	N	Vaccine	Stream
Kampmann et al. (MATISSE) (2)	Phase 3 RCT	Multinational	7,392	Pfizer RSVpreF	Efficacy/safety
Dieussaert et al. (GRACE) (3)	Phase 3 RCT (stopped early)	Multinational	5,328	GSK RSVPreF3-Mat	Efficacy/safety
Madhi et al. (PREPARE) (4)	Phase 3 RCT	Multinational	4,636	Novavax RSV F	Efficacy/safety
Muñoz et al. (20)	Phase 2 RCT	USA	50	Novavax RSV F	Safety/immunogenicity
Simões et al. (7)	Phase 2b RCT	Multinational	406	Pfizer RSVpreF	Safety/immunogenicity
Bebia et al. (19)	Phase 2 RCT	Multinational	213	GSK RSVPreF3	Safety/immunogenicity
Bebia et al. (17)	Phase 3 RCT (high-risk)	Multinational	169	GSK RSVPreF3-Mat	Safety/immunogenicity
Myer et al. (MORISOT) (25)	Phase 3 RCT (HIV+)	South Africa	343	Pfizer RSVpreF	Safety/immunogenicity
Muñoz et al. (10)	RCT (pilot)	USA	35	Wyeth PFP-2	Safety/immunogenicity
Pérez Marc et al. (BERNI) (13)	TNCC	Argentina	505	Pfizer	Effectiveness
McLachlan et al. (Scotland) (12)	Nested CC	Scotland	27,565	Pfizer	Effectiveness
Williams et al. (UK1) (16)	TNCC	UK	537	Pfizer	Effectiveness
O’Hagan et al. (UK2) (14)	TNCC	UK	658	Pfizer	Effectiveness
Rick et al. (CASSATT) (15)	TNCC	USA	274	Pfizer	Effectiveness
Gentile et al. (27)	TNCC	Argentina	187	Pfizer	Effectiveness
Razzini et al. (RIMA) (26)	Nested TNCC	Argentina	surveillance	Pfizer	Effectiveness
Son et al. (21)	Retrospective cohort	USA	2,973	Pfizer	Safety
Solsman et al. (Utah) (9)	Statewide cohort	USA	24,213	Pfizer	Safety
Gabet et al. (France) (23)	National matched cohort	France	29,032	Pfizer	Safety
Michnick et al. (Sentinel) (22)	Surveillance cohort	USA	13,619	Pfizer	Safety
Kitano et al. (TriNetX) (24)	PS-matched cohort	USA	11,265 pairs	Pfizer	Safety
VSD 2025 (abstract) (8)	Matched cohort	USA	14,099 pairs	Pfizer	Safety
Blauvelt et al. (28)	Retrospective cohort	USA	647	Pfizer	Safety
Razai et al. (South London) (11)	Cross-sectional	UK	911	Pfizer	Safety

All observational studies evaluated the licensed Pfizer bivalent RSVpreF vaccine. RCT, randomised controlled trial; TNCC, test-negative case–control; CC, case–control; PS, propensity score.

### Risk of bias in included studies

Risk of bias was low for seven of the nine randomised trials; two were rated as having some concerns: GRACE (3) [early termination affecting the truncated efficacy analysis) and the PFP-2 pilot trial Muñoz et al. (10)], which pre-dated mandatory trial registration). Among the 15 observational studies, 13 were of good methodological quality (Newcastle–Ottawa 8–9 of 9), one was fair (South London (11), 6/9; a single-centre cross-sectional study reporting only unadjusted perinatal comparisons), and one (the Vaccine Safety Datalink report) was available only as a conference abstract and could not be fully appraised (Figure 2). Full domain-level assessments are provided in Supplementary material S5.

**Figure 2:**
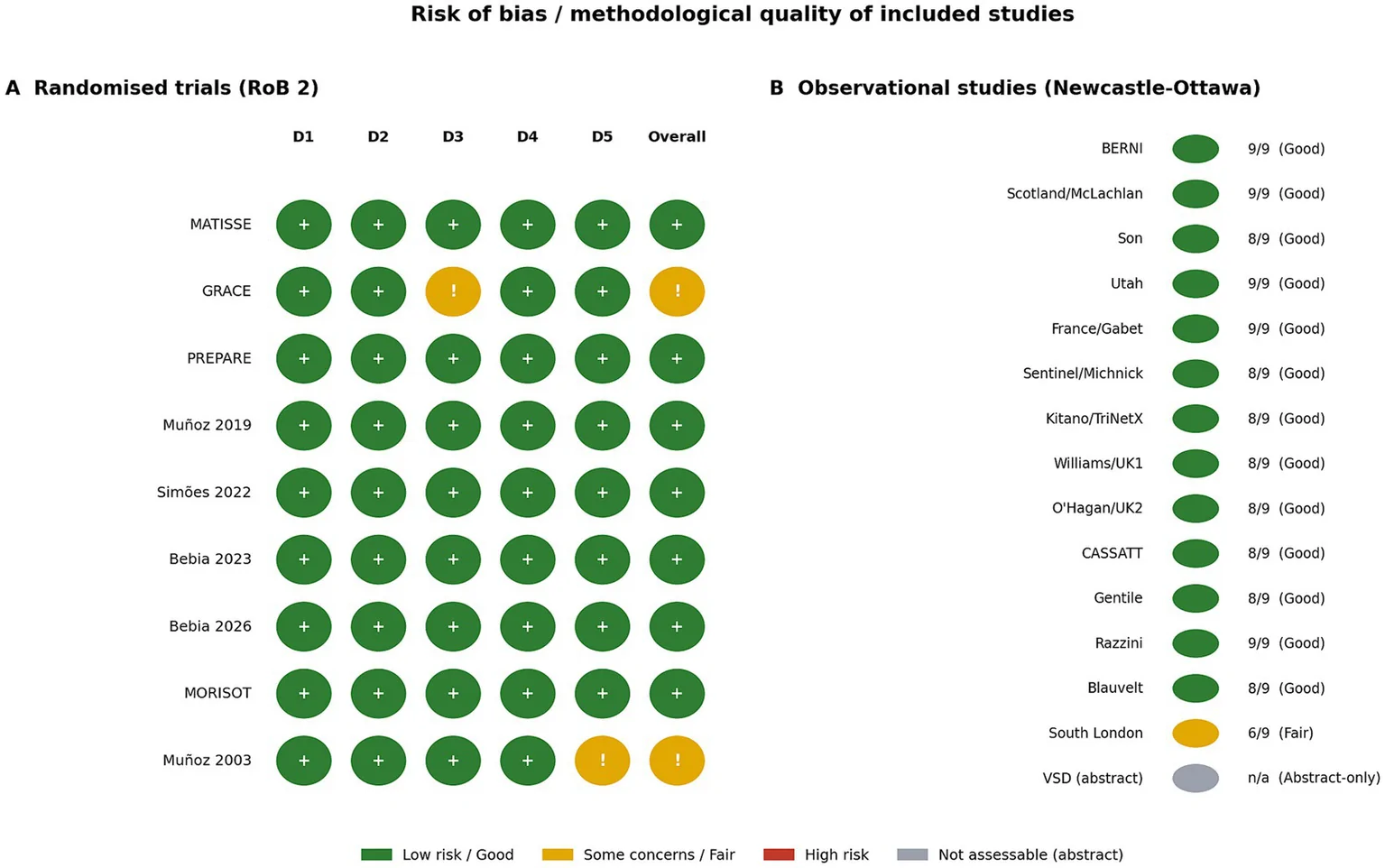
Risk of bias and methodological quality of included studies. **(A)** Randomised trials assessed with RoB 2 (D1 randomisation; D2 deviations; D3 missing outcome data; D4 outcome measurement; D5 selective reporting). **(B)** Observational studies assessed with the Newcastle–Ottawa Scale. Green = low risk/good; amber = some concerns/fair.

An overview of all analyses—contributing studies, design, vaccine platform, outcome, follow-up window, and effect measure—is provided in Table 2, which serves as a roadmap to the outcome-specific results that follow.

**Table 2:** Summary of analyses: Contributing studies, study design, vaccine platform, outcome, follow-up window, and effect measure.

Analysis (outcome)	Design	Vaccine platform (s)	Contributing studies	Follow-up/window	Effect measure	Estimate (95% CI)
Effectiveness, RSV hospitalisation, 0–3 mo	Observational (case–control: 3 test-negative + Scotland population-based)	Pfizer	Scotland, BERNI, O’Hagan, CASSATT	≤90 days	VE (from adjusted OR)	79.7% (73.9–84.2)
Effectiveness, RSV hospitalisation, 0–6 mo	Observational (test-negative)	Pfizer	BERNI, Williams, O’Hagan	≤180 days	VE (from adjusted OR)	68.1% (57.1–76.3)
Efficacy, severe RSV-LRTI—licensed Pfizer (primary)	RCT	Pfizer	MATISSE	180 d (and 90 d)	VE	70.0% (50.6–82.5); 82.4% at 90 d
Efficacy, severe RSV-LRTI—cross-platform (exploratory)	RCT	Pfizer, GSK, Novavax	MATISSE, GRACE, PREPARE	90–180 d (differs)	VE	65.1% (49.1–76.1)
Efficacy, medically attended RSV-LRTI—cross-platform (exploratory)	RCT	Pfizer, GSK, Novavax	MATISSE, GRACE, PREPARE	90–180 d (differs)	VE	51.5% (36.3–63.1)
Preterm birth—randomised	RCT	Pfizer, GSK, Novavax	7 trials (MATISSE, GRACE, PREPARE, Bebia et al. (17), MORISOT, Simões, Muñoz)	To birth	RR (Mantel–Haenszel)	1.17 (1.03–1.32)
Preterm birth—observational	Observational (cohort)	Pfizer	Utah, Sentinel, Son, France, Kitano	To birth	Adjusted OR/IRR/ARR	0.90 (0.82–0.99)
Hypertensive disorders of pregnancy—observational (by adjustment)	Observational (cohort)	Pfizer	Son, Sentinel (limited); France, Kitano (thorough)	Pregnancy	Adjusted OR/IRR/ARR	Limited-adj 1.13 (1.03–1.25); thorough-adj 1.01 (0.96–1.07)

The exploratory cross-platform efficacy pools combine three different vaccines with differing severe disease definitions and follow-up windows and are interpreted with caution; the licensed Pfizer (MATISSE) estimate is the primary efficacy reference. VE, vaccine effectiveness/efficacy; OR, odds ratio; IRR, incidence rate ratio; ARR, adjusted rate ratio; RR, risk ratio.

### Effectiveness against infant RSV-associated hospitalisation (0–3 months)

Four independent real-world studies estimated the effectiveness of maternal RSVpreF vaccination against RSV-associated hospitalisation in infants during the first 3 months of life: a national population-based study from Scotland (McLachlan (12)), the multicentre test-negative BERNI study from Argentina (Pérez Marc (13)), the UK Season 2 PERUKI/BronchStart study (O’Hagan (14)), and the single-centre CASSATT study from the United States (Rick (15)). These four studies were mutually independent (verified by study period, geography, and, for overlapping clusters, participating hospital sites) and each reported effectiveness for a comparable 0–3-month (≤90-day) window. Because all four used a case–control design (three test-negative and one, the Scottish national study, a population-based matched case–control), effectiveness was derived from adjusted odds ratios and pooled using the pre-specified random-effects method (see Methods). All observational studies evaluated the licensed Pfizer bivalent RSVpreF vaccine. In each study, RSV was laboratory-confirmed from a respiratory specimen, predominantly by real-time reverse transcriptase PCR, with antigen or immunofluorescence testing accepted in some studies, rather than by clinical diagnosis; RSV-positive infants were classified as cases and RSV-negative infants (or, in the Scottish national study, gestational age- and birth week-matched population controls) as controls, so all RSV-associated hospitalisation outcomes were laboratory-confirmed.

Individual estimates ranged from 67.6% to 82.2% and were closely consistent. The pooled vaccine effectiveness against infant RSV-associated hospitalisation in the first three months of life was 79.7% (95% CI 73.9%–84.2%), with no statistical heterogeneity (*I*^2^ = 0%; Q = 2.12, *df* = 3). Scotland contributed the greatest weight (55.9%), followed by BERNI (19.5%), O’Hagan (13.6%) and CASSATT (11.0%). The absence of heterogeneity across four studies from different countries and health systems indicates a consistent, reproducible effectiveness signal (Figure 3). Although this pool combined three test-negative studies with one population-based matched case–control (Scotland), a leave-one-out analysis that included the removal of Scotland left the estimate within a narrow range (76.1%–80.9%), indicating that the design heterogeneity did not materially affect the pooled effectiveness. Full study-level outcome data for all analyses are provided in Supplementary material S6.

**Figure 3:**
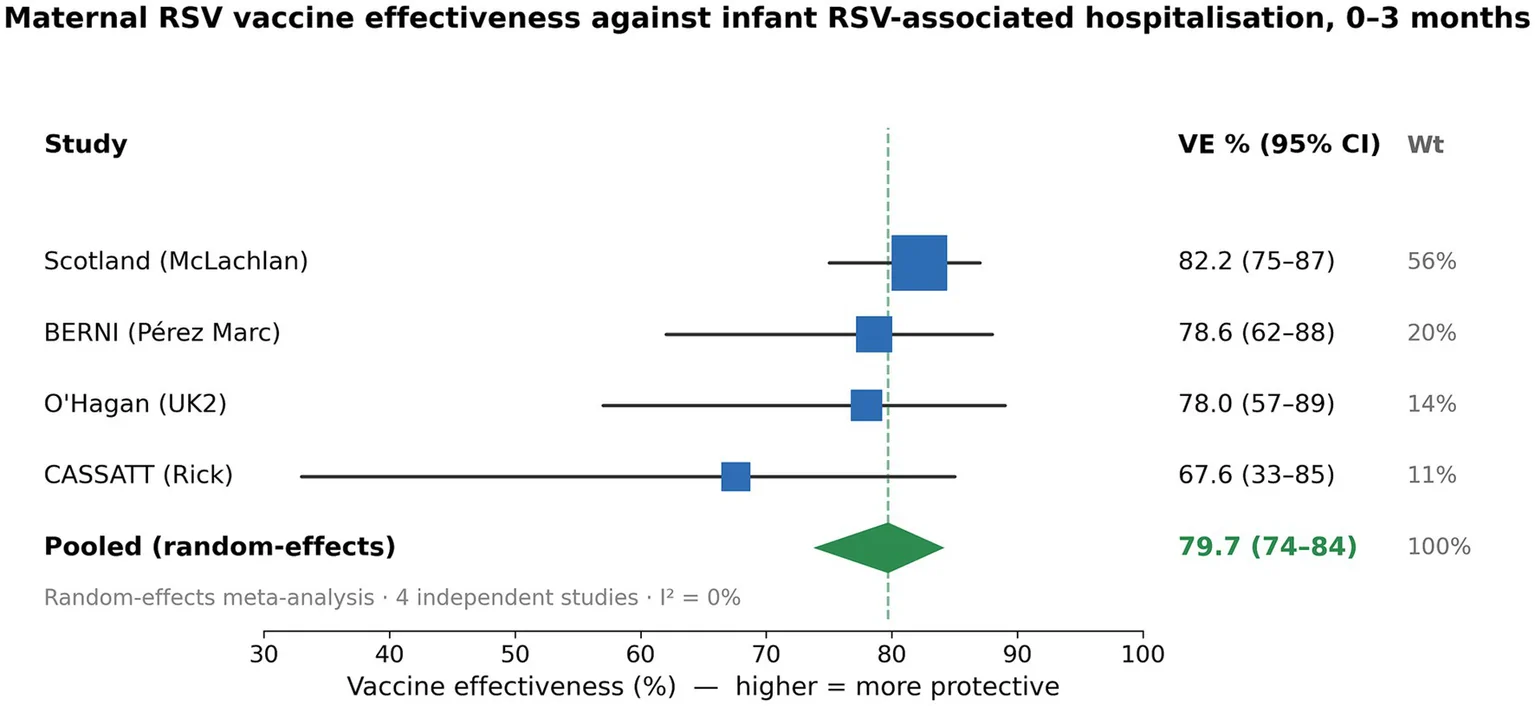
Forest plot of maternal RSV vaccine effectiveness against infant RSV-associated hospitalisation (0–3 months), random-effects meta-analysis of four independent case–control studies (three test-negative and one, the Scottish National Study, population-based). Pooled estimate 79.7% (95% CI 73.9–84.2%); *I*^2^ = 0%. Analytic sample: 4 studies, 5,143 infants [1,040 RSV-positive cases and 4,103 controls (test-negative or, for the Scottish National Study, gestational age-matched population controls)]. VE, vaccine effectiveness; CI, confidence interval; Wt, study weight (percentage contribution to the pooled estimate); *I*^2^, proportion of total variation across studies attributable to heterogeneity.

### Effectiveness through 6 months

Effectiveness through the first six months of life was estimated from three independent studies reporting a 0–6-month window with a consistent definition of effective vaccination (>14 days before delivery): BERNI (Argentina), Williams (16)/UK1 (UK Season 1), and O’Hagan/UK2 (UK Season 2). Williams was used as the UK Season-1 contributor for this window because the Scottish national study reported effectiveness only through ≤90 days; as it does not enter the 6-month pool, no double-counting arises.

The pooled effectiveness against infant RSV-associated hospitalisation through 6 months of age was 68.1% (95% CI 57.1%–76.3%), with no heterogeneity (*I*^2^ = 0%; Q = 0.87, *df* = 2) (Figure 4). Effectiveness was thus lower over the wider 0-6-month age window than over the 0–3-month window (79.7%), while remaining substantial (~68%) through 6 months.

**Figure 4:**
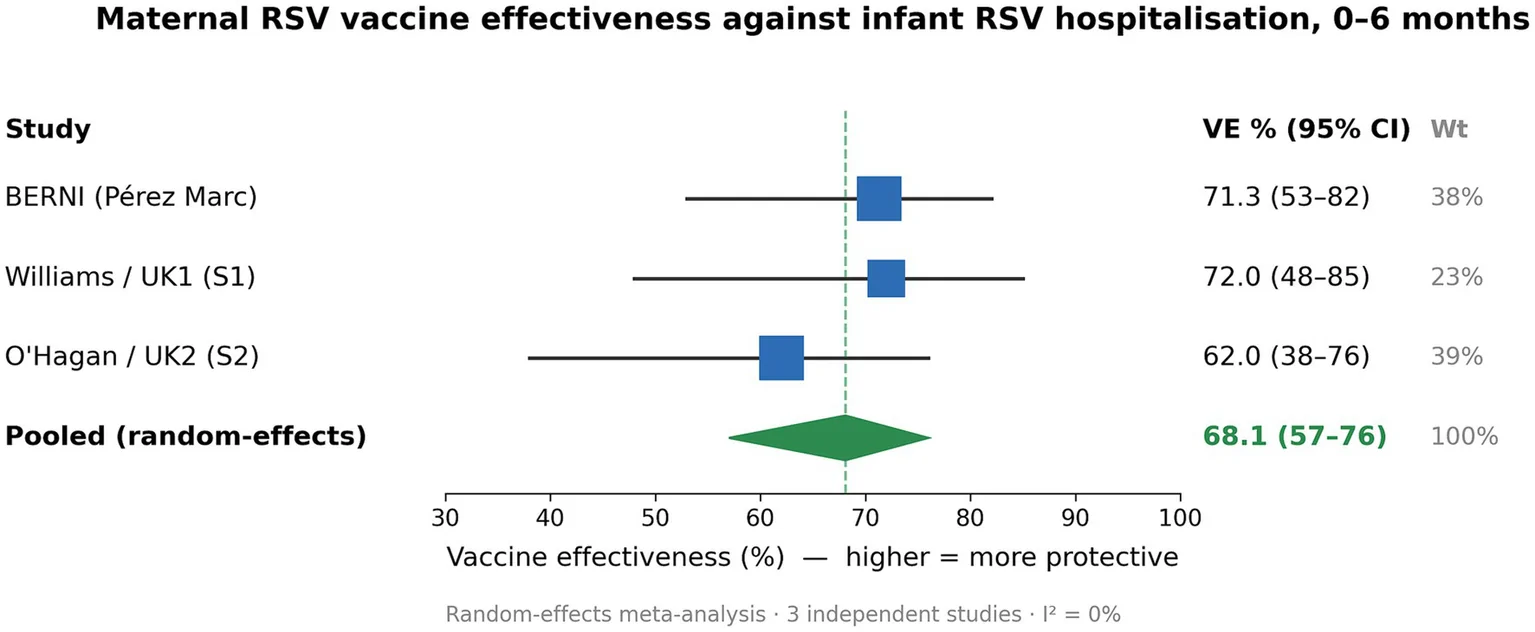
Forest plot of maternal RSV vaccine effectiveness against infant RSV-associated hospitalisation through 6 months (three independent studies); pooled 68.1% (95% CI 57.1%–76.3%); *I*^2^ = 0%. Analytic sample: 3 studies, 1,700 infants (1,079 RSV-positive cases and 621 test-negative controls). VE, vaccine effectiveness; CI, confidence interval; Wt, study weight (percentage contribution to the pooled estimate); *I*^2^, proportion of total variation across studies attributable to heterogeneity.

### Neonatal and earliest-infancy outcomes

None of the included studies reported RSV-associated hospitalisation over a discrete 0–28-day neonatal window; the finest early-life resolution reported was 0–90 days (0–3 months), which encompasses the neonatal period, so a neonatal-specific pooled estimate could not be computed separately. Within this earliest window, protection was greatest: pooled effectiveness against infant RSV-associated hospitalisation was 79.7% (95% CI 73.9–84.2) at 0–3 months, higher than at 0–6 months (68.1%, 95% CI 57.1–76.3), and in the preterm-infant subgroup, effectiveness remained high (Scotland (12), vaccine effectiveness 89.9%, 95% CI 55.3–97.7; a second study, CASSATT (15), reported a concordant but highly imprecise preterm-subgroup estimate, 63.5%, 95% CI − 101.7 to 95.4). RSV-associated neonatal and infant deaths were too infrequent for meta-analysis, and no death was attributed to maternal vaccination. All-cause neonatal death was not increased: the only numerical imbalance, in the GSK trial (GRACE; RR 2.16, 95% CI 0.62–7.56), was non-significant and consistent with that vaccine’s excess prematurity, and the high-risk GSK phase-3 trial Bebia et al. (17) reported one neonatal death in each group.

### Efficacy against infant RSV-LRTI (randomised controlled trials)

Three phase 3 randomised trials reported efficacy against infant RSV-associated lower respiratory tract illness: MATISSE (2) (Pfizer bivalent RSVpreF), GRACE (GSK RSVPreF3-Mat), and PREPARE (4) (Novavax RSV F nanoparticle). These trials are not directly comparable: they evaluated three different vaccines and, importantly, used different definitions of severe disease and different follow-up windows. MATISSE and GRACE both defined severe RSV-LRTI by clinical severity criteria assessed over 180 days and yielded closely concordant efficacy (MATISSE 70.0, 95% CI 50.6%–82.5%, through 180 days and 82.4% through 90 days (18); GRACE 69.0, 95% CI 33%–86%, through 180 days). PREPARE instead reported a different outcome—RSV-LRTI with severe hypoxaemia—assessed only over 90 days, with a lower efficacy of 48.3% (95% CI − 8.2 to 75.3%). Because MATISSE evaluates the only licensed maternal vaccine and is matched by GRACE on both outcome definition and follow-up window, it is taken as the primary source of severe disease efficacy (≈69% through 180 days; 81.8% through 90 days); PREPARE’s estimate is not directly comparable and is reported separately. In all three randomised trials, the RSV-associated lower respiratory tract illness efficacy endpoints required laboratory confirmation of RSV by reverse transcriptase PCR.

For completeness, a random-effects meta-analysis pooling all three trials gave a summary efficacy against severe RSV-LRTI of 65.1% (95% CI 49.1%–76.1%) and against any medically attended RSV-LRTI of 51.5% (95% CI 36.3%–63.1%), with no statistical heterogeneity (*I*^2^ = 0%). Because this pool spans three different vaccines, two definitions of severe disease, and two follow-up windows, it is reported only as an exploratory, approximate cross-platform summary and is interpreted with caution; the licensed Pfizer vaccine (MATISSE) is the primary source of severe disease efficacy (Figure 5). A structured head-to-head comparison of the three vaccine platforms—integrating efficacy, real-world effectiveness, immunogenicity, and the preterm-birth signal—is presented in Table 3.

**Figure 5:**
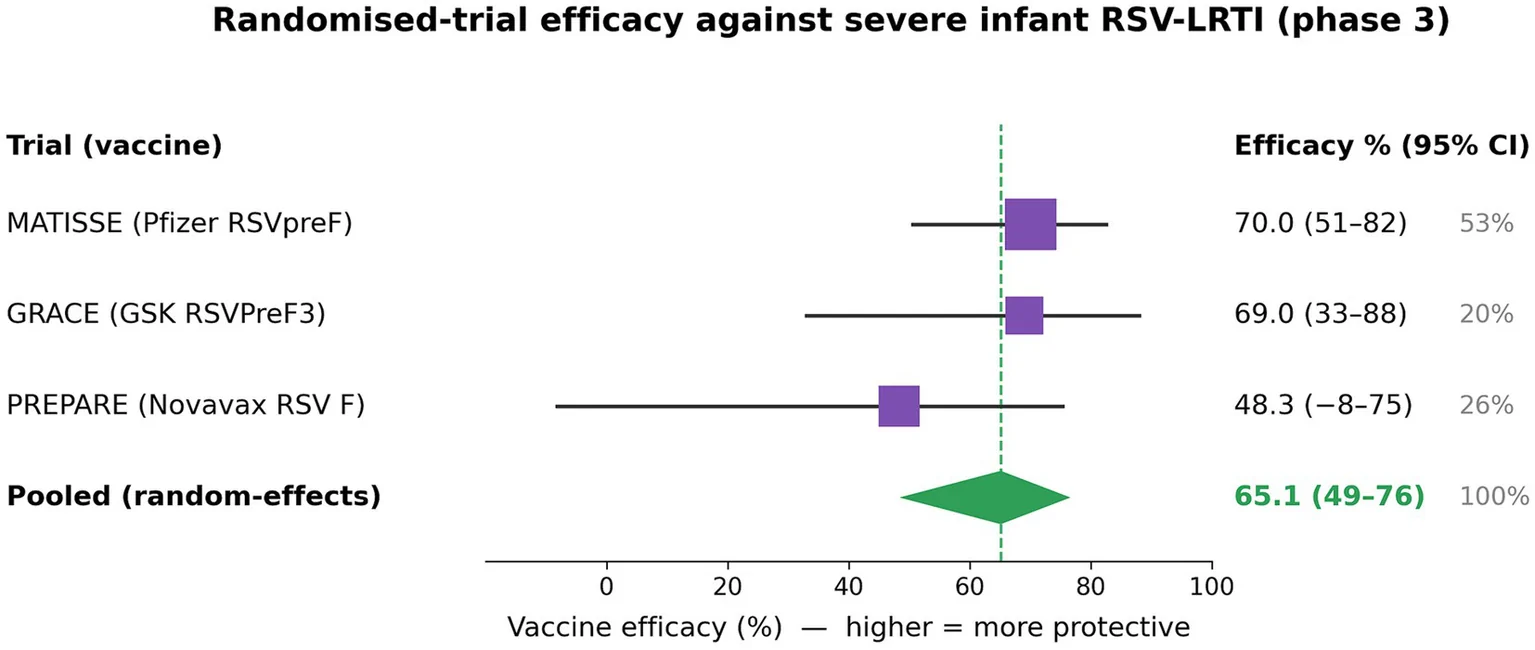
Randomised-trial efficacy against severe infant RSV-LRTI. MATISSE (Pfizer) and GRACE (GSK) define severe RSV-LRTI by clinical severity criteria over 180 days (70.0% and 69.0%, respectively); PREPARE (Novavax) reports severe hypoxaemia over 90 days (48.3%) and is not directly comparable. The random-effects pooled estimate across all three platforms (65.1, 95% CI 49.1%–76.1%; *I*^2^ = 0%) is presented only as an exploratory, approximate cross-platform summary, given the differing vaccines, outcome definitions, and follow-up windows, and is not the basis for the efficacy–effectiveness comparison. CI, confidence interval; Wt, study weight (percentage contribution to the pooled estimate); *I*^2^, proportion of total variation across studies attributable to heterogeneity; RSV-LRTI, RSV lower respiratory tract infection.

**Table 3:** Comparison of the three maternal RSV vaccine platforms, integrating randomised efficacy, real-world effectiveness, immunogenicity, and the preterm-birth safety signal.

Attribute	Pfizer bivalent RSVpreF	GSK RSVPreF3-Mat	Novavax RSV F nanoparticle
Vaccine (trade name)	Bivalent RSVpreF (Abrysvo)	RSVPreF3-Mat	RSV F nanoparticle (ResVax)
Antigen/platform	Bivalent prefusion F (RSV-A + B); unadjuvanted	Prefusion F (RSVPreF3); unadjuvanted	F-protein nanoparticle; ± aluminium phosphate
Key trials [ref]	MATISSE (2); Simões et al. (7); MORISOT (25)	GRACE (3); Bebia et al. (19); Bebia et al. (17)	PREPARE (4); Muñoz et al. (20)
Vaccination window	Licensed 32–36 weeks (trials 24–36 wk)	Trials ~24–34 weeks	Trials ~28–36 weeks
Efficacy vs. severe RSV-LRTI	70.0% (50.6–82.5) at 180 d; 82.4% at 90 d	69.0% (33–86) at 180 d	48.3% (−8.2–75.3) vs. severe hypoxaemia at 90 d
Primary efficacy endpoint	Met → licensed	Efficacy demonstrated; development halted	Not met (failed primary endpoint)
Real-world effectiveness (infant RSV hospitalisation)	79.7% (0–3 mo); 68.1% (0–6 mo)—all real-world data pertain to this vaccine	None (not deployed)	None (not deployed)
Transplacental transfer ratio (Supplementary Table S10)	1.41–2.10	1.33–1.90	1.04–1.17 (least efficient transfer)
Preterm-birth signal, RR (95% CI)	1.20 (0.98–1.46)—not robustly significant	1.37 (1.08–1.75)—significant excess	0.95 (0.75–1.21)—null
Regulatory/development status	Licensed for maternal use (2023); recommended 32–36 weeks	Not advanced; trial terminated early for preterm-birth imbalance	Not advanced; failed primary efficacy

RR, risk ratio; d, days; wk, weeks. Efficacy definitions and follow-up windows differ across trials and are not directly comparable (see Results and Discussion); the pooled cross-platform efficacy estimate is reported in the text. Real-world effectiveness pertains exclusively to the licensed Pfizer vaccine (all 15 observational studies). Transplacental transfer ratios are summarised in Supplementary Table S10. Preterm birth risk ratios are the pivotal trial values (MATISSE, GRACE, PREPARE).

### Vaccine immunogenicity (narrative summary)

Although vaccine immunogenicity was not a pre-specified outcome of this review and was not formally synthesised, for completeness we note that the included randomised trials consistently reported robust maternal antibody responses (broadly of the order of eight- to nineteen-fold over pre-vaccination levels), transplacental transfer with cord-blood-to-maternal antibody transfer ratios generally at or above one (approximately 1.0 for the Novavax F-nanoparticle vaccine and approximately 1.3 to 2.1 for the prefusion-F vaccines), and infant antibody levels that were highest at birth and declined over the first months of life (4, 7, 17, 19). Because the trials used different neutralisation assays and reporting units, these values are not directly comparable across platforms and are reported narratively only (Supplementary material S10).

### Safety: preterm birth

Preterm birth was examined separately in randomised trials and observational studies. Across the randomised trials, the pooled risk ratio (Mantel–Haenszel) was 1.17 (95% CI 1.03–1.32; Cochran–Mantel–Haenszel *χ*^2^ = 6.25, *p* = 0.012; *I*^2^ = 8%), indicating a small but statistically significant increase, driven principally by the GSK trial (GRACE; RR 1.37, 95% CI 1.08–1.75) and a non-significant excess in MATISSE (RR 1.20, 95% CI 0.98–1.46); the Novavax trial (PREPARE) was null (RR 0.95, 95% CI 0.75–1.21). The pooled estimate comprised seven randomised trials: the three phase 3 efficacy trials above accounted for approximately 92% of the weight, and four smaller trials Bebia et al. (17), RR 0.92, 95% CI 0.50–1.69; MORISOT, RR 1.12, 95% CI 0.55–2.28; Simões et al. (7), RR 3.36, 95% CI 0.45–25.17, from a small adverse-event-coded count of 14/325 vs. 1/78; Muñoz et al. (20), 1/22 vaccine vs. 0/28 placebo, a zero-event comparator arm together contributed the remaining ~8% without materially changing the estimate. All seven trials are shown in Figure 6. A further GSK RSVPreF3-Mat phase-2 trial in healthy pregnant women (Bebia et al. (19), 213 participants) did not report a poolable preterm estimate and was therefore not included in this pool, but recorded no vaccine-related pregnancy or neonatal adverse events of special interest.

**Figure 6:**
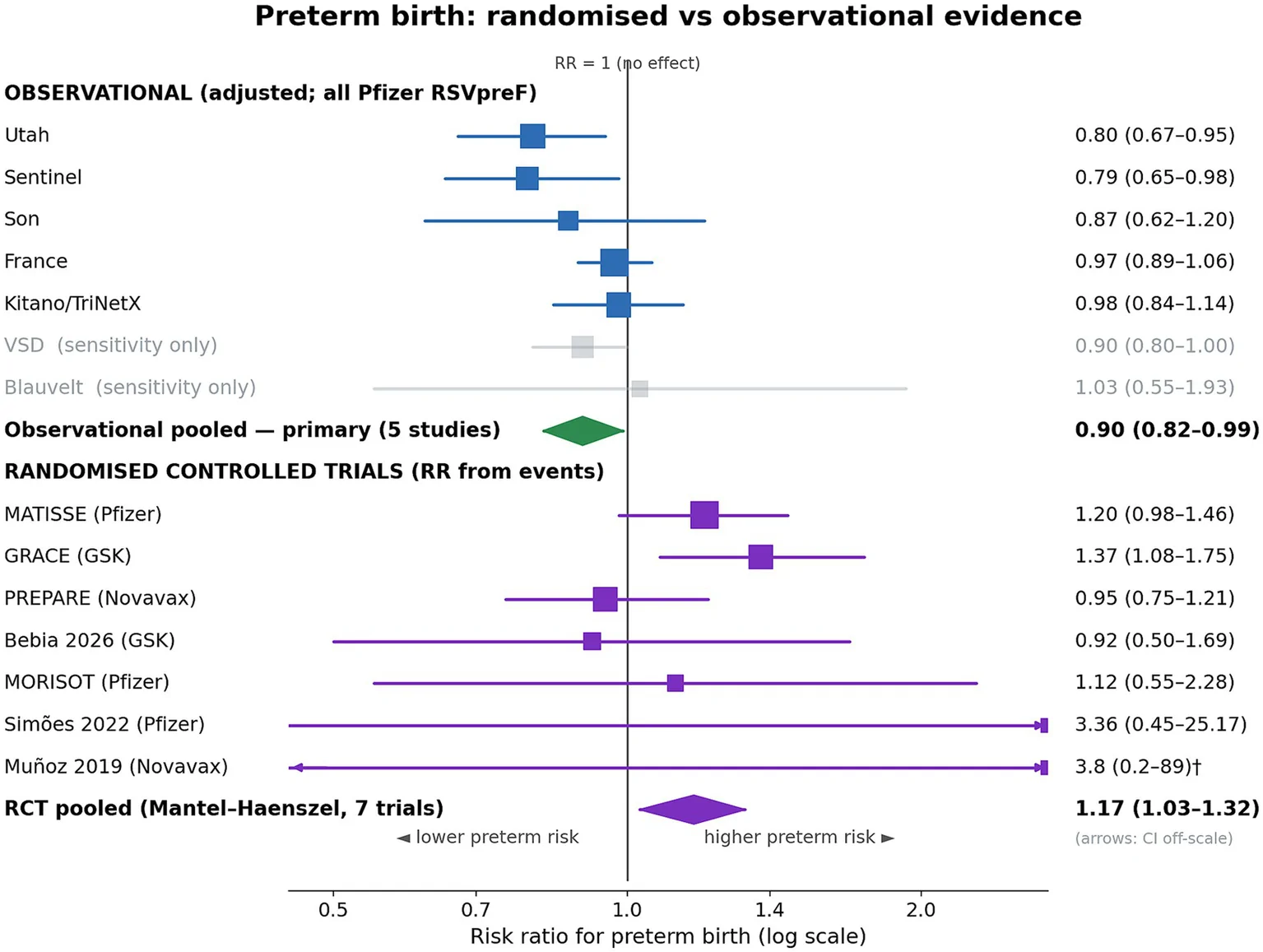
Preterm birth—randomised versus observational evidence. Pooled randomised *RR* 1.17 (95% *CI* 1.03–1.32; Mantel–Haenszel); pooled observational adjusted ratio 0.90 (95% *CI* 0.82–0.99). The opposite directions are consistent with residual healthy vaccinee confounding in the observational studies. Analytic samples: Randomised pool, 7 trials (18,071 infants); observational pool, 5 studies (53,534 RSVpreF-vaccinated pregnancies plus matched/comparator pregnancies). *RR*, risk ratio; *CI*, confidence interval; RCT, randomised controlled trial; VSD, Vaccine Safety Datalink; RSVpreF, RSV prefusion F. Blue = observational; purple = randomised; diamonds = pooled estimates. Arrows indicate confidence intervals extending off-scale.

**Figure 7:**
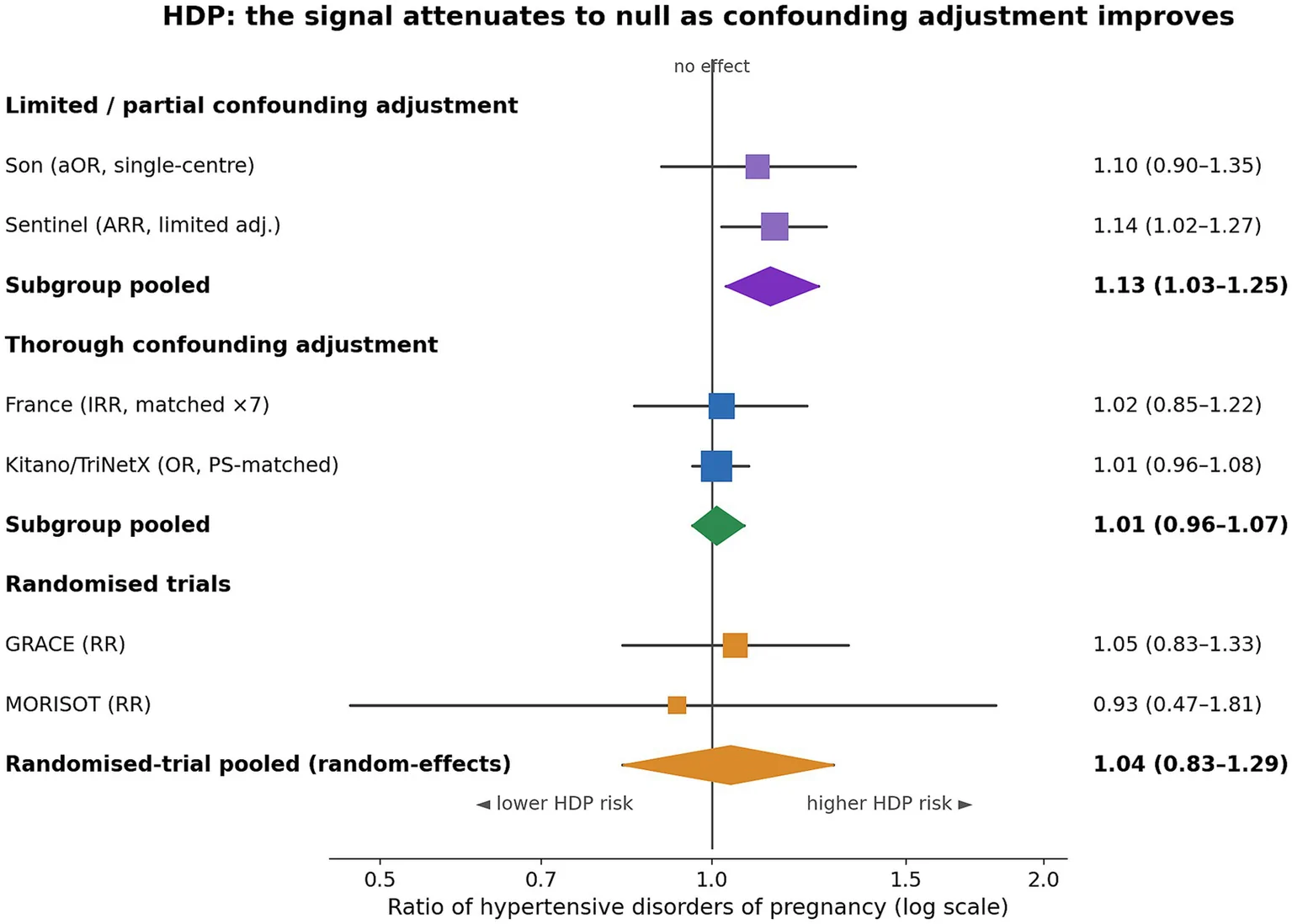
HDP: Observational studies stratified by confounding adjustment, plus two randomised trials. Limited-adjustment pooled 1.13 (95% CI 1.03–1.25; *I*^2^ = 0%); thorough-adjustment pooled 1.01 (95% CI 0.96–1.07; *I*^2^ = 0%); both randomised trials yielded null results (GRACE 1.05, 95% CI 0.83–1.33; MORISOT 0.93, 95% CI 0.47–1.81). Analytic samples: 4 Observational studies (50,801 RSVpreF-vaccinated pregnancies plus comparators) plus two randomised trials (GRACE, 5,233 infants; MORISOT, 343 women). Sentinel contributes composite concurrent and historical comparator groups. MORISOT’s hypertensive disorder estimate is a composite of gestational hypertension, pre-eclampsia, and postpartum hypertension. HDP, hypertensive disorders of pregnancy; aOR, adjusted odds ratio; OR, odds ratio; ARR, adjusted rate ratio; IRR, incidence rate ratio; RR, risk ratio; PS, propensity score; CI, confidence interval.

In contrast, the pooled adjusted estimate from five observational studies of the licensed Pfizer vaccine was 0.90 (95% CI 0.82–0.99; *I*^2^ = 41%), pointing in the opposite direction to the randomised estimate (Figure 6).

### Safety: hypertensive disorders of pregnancy (HDP)

Six studies reported HDP (four observational and two randomised). Pooling the four observational estimates gave a ratio of 1.05 (95% CI 0.99–1.12) with moderate heterogeneity (*I*^2^ = 25%). In an exploratory, post-hoc stratification, the heterogeneity was explained almost entirely by the degree of confounding control: in studies with limited adjustment (Son (21); and Sentinel (22), which its authors noted had limited confounding control), the pooled estimate was modestly elevated but homogeneous (1.13, 95% CI 1.03–1.25; *I*^2^ = 0%). Conversely, in thoroughly adjusted studies (France (23), matched 1:1 on seven factors; and Kitano (24), propensity score matched on baseline hypertension, diabetes, obesity, and prior preterm labour), it was null and consistent (1.01, 95% CI 0.96–1.07; *I*^2^ = 0%). Both randomised trials reporting HDP were null (GRACE (3) RR 1.05, 95% CI 0.83–1.33; MORISOT (25) RR 0.93, 95% CI 0.47–1.81, a composite of gestational hypertension, pre-eclampsia, and postpartum hypertension).

### Safety: other obstetric and neonatal outcomes

Other obstetric and neonatal outcomes showed no increase (Table 4; Supplementary material S11). Low birth weight was not significantly increased (pooled RR 1.09, 95% CI 0.93–1.28; *I*^2^ = 6%), paralleling the preterm findings as a downstream consequence of prematurity. Small-for-gestational-age births showed no effect (pooled 1.01, 95% CI 0.97–1.06; *I*^2^ = 0%). Single-study outcomes were also reassuring: stillbirth was not increased (PREPARE 0.87; France 0.77), congenital anomalies were not increased (MATISSE 0.85), and neonatal death was numerically higher in the GSK trial (GRACE 2.16, 95% CI 0.62–7.56) but non-significant and consistent with excess prematurity. On complete-panel review, additional outcomes (gestational diabetes, placental abruption, IUGR, IUFD, caesarean delivery, postpartum haemorrhage, cardiovascular events, LGA, and further stillbirth/LBW analyses) were uniformly null, with no maternal Guillain–Barré cases; no new independent safety signal emerged. For completeness, two further pre-specified secondary outcomes are accounted for explicitly. RSV-associated intensive care unit admission was not reported as a separable, poolable outcome by the included studies and could not be meta-analysed; the severity spectrum was instead captured by the severe RSV-LRTI and hospitalisation analyses reported above. RSV-associated infant mortality was rarely reported as a distinct outcome and involved too few events for synthesis; the only mortality data, the numerically higher neonatal-death count in the GSK trial noted above, was non-significant and attributable to excess prematurity, and no study reported an excess of infant deaths with maternal vaccination. A consolidated comparison of all maternal and birth outcomes in vaccinated versus unvaccinated or placebo pregnancies is presented in Table 4.

**Table 4:** Maternal and birth (obstetric and neonatal) outcomes in vaccinated versus unvaccinated or placebo pregnancies: Pooled or representative estimates.

Outcome	Evidence (design; studies)	Estimate (95% CI)	Interpretation
Preterm birth (<37 wk)	Randomised (7 trials)	RR 1.17 (1.03–1.32)	Statistically significant small increase overall (CI excludes 1), driven by the withdrawn GSK vaccine; not robustly significant for the licensed Pfizer vaccine
Preterm birth (<37 wk)	Observational (5 studies)	0.90 (0.82–0.99)	Statistically significant apparent reduction (CI excludes 1); biologically implausible and consistent with healthy vaccinee confounding
Low birth weight	Randomised (MATISSE, GRACE)	RR 1.09 (0.93–1.28)	Not significantly increased (95% CI crosses 1); tracks preterm birth
Small-for-gestational-age	Observational (France, VSD)	1.01 (0.97–1.06)	No significant effect (95% CI crosses 1)
Stillbirth	Randomised (PREPARE)	0.87 (0.38–1.97)	No significant difference (95% CI crosses 1)
Stillbirth	Observational (France)	0.77 (0.45–1.32)	No significant difference (95% CI crosses 1)
Hypertensive disorders of pregnancy	Observational (4 studies)	All 1.05 (0.99–1.12); thorough-adjustment 1.01 (0.96–1.07)	Null after adequate adjustment (thorough-adjustment CI crosses 1); the limited-adjustment signal reflects residual confounding
Gestational hypertension	Observational (Kitano)	1.11 (1.04–1.19)	Statistically significant small excess (CI excludes 1) in limited-adjustment data; likely residual confounding
Pre-eclampsia	Observational (Kitano; France)	0.88 (0.81–0.95); 1.02 (0.85–1.22)	No consistent increase (one estimate significantly lower, the other crosses 1)
Gestational diabetes	Observational (Kitano)	0.98 (0.90–1.06)	No significant effect (95% CI crosses 1)
Caesarean delivery	Observational (France)	1.00 (0.96–1.03)	No significant effect (95% CI crosses 1)
Postpartum haemorrhage	Observational (France)	1.03 (0.97–1.10)	No significant effect (95% CI crosses 1)
Congenital anomalies	Randomised (MATISSE)	0.85 (0.70–1.04)	No significant difference (95% CI crosses 1)
NICU admission	Observational (Utah)	0.77 (0.65–0.91)	Statistically significant apparent reduction (CI excludes 1); likely healthy vaccinee confounding
Neonatal death	Randomised (GRACE)	2.16 (0.62–7.56)	Non-significant (95% CI crosses 1); downstream of the GSK vaccine’s excess prematurity

Estimates are random-effects pooled where two or more comparable studies were available; otherwise, representative single-study estimates are provided. Observational estimates are adjusted. Different adjusted ratio measures (adjusted odds ratios, risk ratios, incidence rate ratios, and adjusted rate ratios) were treated as approximately equivalent. Forest plots for preterm birth and hypertensive disorders are shown in Figures 6, 7; other obstetric and neonatal outcomes are shown in Supplementary material. RR, risk ratio; NICU, neonatal intensive care unit. Statistical significance is judged by whether the 95% confidence interval excludes 1; an interval that crosses 1 indicates no statistically significant difference.

### Safety: reactogenicity

Reactogenicity (solicited local and systemic reactions within 7 days) was assessed in the randomised trials and summarised narratively. Local injection-site reactions, predominantly pain, were more common with the vaccine than with placebo (for example, 40.7% versus 9.9% in PREPARE; 59% versus 4% in the Novavax phase 2 trial; 65% versus 13% in the PFP-2 trial) and were mostly mild to moderate and self-limiting. Systemic reactions (fever, fatigue, headache, myalgia) were generally similar between groups, and no reactogenicity-related serious adverse events or grade-3 excess were reported in any trial. Reactogenicity was mild to moderate in pregnant people living with HIV (MORISOT). The profile is as expected for a non-live recombinant protein subunit vaccine. Full solicited-reaction data are provided in Supplementary material S8.

### Sensitivity analyses

Pre-specified sensitivity analyses tested robustness. For effectiveness, adding the overlapping regional studies reserved from the primary analysis (Razzini (26), Gentile (27)) left the estimate essentially unchanged (0–3 months 79.7% → 79.7%; 0–6 months 68.1% → 67.9%), and leave-one-out analysis produced a narrow range (76.1–80.9% at 0–3 months) even when the most heavily weighted study (Scotland) was removed. Fixed-effect and random-effects models gave identical results throughout (*I*^2^ = 0%). Of the pre-specified subgroup analyses, those by vaccine type (efficacy and preterm birth) and by study design (randomised versus observational throughout, and by degree of confounding adjustment for hypertensive disorders of pregnancy) were feasible and are reported above; the influence of gestational age at vaccination is captured by the product-specific and dosing window analyses of preterm birth. Subgroup analyses by preterm versus term infant status and by geographic region could not be performed, because too few studies reported the necessary strata (for the preterm-versus-term comparison, only two effectiveness studies—Scotland (12) and CASSATT (15)—reported a preterm stratum, and only the former had usable precision), and are therefore not reported.

For efficacy against severe RSV-LRTI, restricting to the licensed Pfizer vaccine (MATISSE, 70.0%) or excluding the unsuccessful Novavax trial (69.7%) gave estimates consistent with, and slightly higher than, the full three-vaccine pool (65.1%).

The preterm-birth sensitivity analysis was the most informative. The significant pooled randomised risk ratio (Mantel–Haenszel 1.17, 95% CI 1.03–1.32) was not robust: excluding the GSK trial reduced it to a non-significant 1.10 (95% CI 0.95–1.27). For the licensed Pfizer vaccine, the increase was small and not robustly significant: the pivotal MATISSE trial (1.20, 95% CI 0.98–1.46) and the two Pfizer trials with dedicated birth outcome analyses (MATISSE and MORISOT; 1.19, 95% CI 0.99–1.44) were both non-significant, whereas the three-trial Pfizer pool was only marginally significant (1.21, 95% CI 1.00–1.46), an estimate that hinged on the small, adverse-event-coded preterm counts of the exploratory phase-2b trial Simões et al. (7); every Pfizer-restricted confidence interval extended to or below the null. The significant preterm signal is therefore driven principally by the withdrawn GSK vaccine; for the licensed Pfizer vaccine, a small increase is possible but not statistically confirmed. No single outcome included 10 or more studies (the largest pooled analysis comprised seven studies), so publication bias could not be formally assessed by funnel plot or Egger’s test; the possibility of small-study effects therefore cannot be excluded and is acknowledged as a limitation. The observational preterm pool was likewise robust: adding back the two studies reserved on quality grounds—the abstract-only Vaccine Safety Datalink report (8) and the uptake-focused Blauvelt cohort (28)—left the primary five-study estimate (0.90, 95% CI 0.82–0.99) essentially unchanged (0.91, 95% CI 0.86–0.97).

### Certainty of evidence (GRADE)

The certainty of evidence was rated for each key outcome using GRADE (Table 5). Effectiveness against RSV-associated hospitalisation was of moderate certainty at both 0–3 months (79.7%) and 0–6 months (68.1%): although derived from observational studies, the estimates were upgraded for a large magnitude of effect and supported by complete consistency, precision, and low risk of bias. Efficacy against severe RSV-LRTI was moderate, downgraded once for indirectness (three vaccine platforms with differing severe disease definitions and follow-up windows). Preterm birth was rated as moderate, downgraded once for indirectness, the significant signal being product-specific (attributable to the withdrawn GSK vaccine; not robustly significant for the licensed Pfizer vaccine) and gestational window-dependent. HDP was of very low certainty (serious inconsistency and risk of bias), reflecting residual confounding. Other outcomes were reassuring, and reactogenicity was summarised narratively. The full GRADE Summary of Findings is presented in Table 5 and Supplementary material S9.

**Table 5:** Summary of findings (GRADE).

Outcome	Studies (design)	Pooled effect (95% CI)	Certainty
RSV hospitalisation, 0–3 mo	4 observational (case–control)	VE 79.7% (73.9–84.2)	Moderate
RSV hospitalisation, 0–6 mo	3 observational (TN)	VE 68.1% (57.1–76.3)	Moderate
Efficacy, severe RSV-LRTI	3 RCTs	VE 65.1% (49.1–76.1)	Moderate
Preterm birth	7 RCTs pooled	RR 1.17 (1.03–1.32)*	Moderate
Hypertensive disorders	4 observational	1.05 (0.99–1.12)	Very low
Other obstetric/neonatal	RCT + obs	All null/reassuring	Moderate

TN = test-negative; VE = vaccine effectiveness/efficacy. *Preterm signal is product-specific (not robustly significant for the licensed Pfizer vaccine) and mitigated by the 32–36-week dosing window.

## Discussion

This systematic review and meta-analysis integrated randomised and real-world evidence on maternal respiratory syncytial virus (RSV) vaccination for the prevention of infant lower respiratory tract infection. Maternal RSVpreF vaccination was highly effective against infant RSV-associated hospitalisation, with a pooled real-world effectiveness of 79.7% (95% CI 73.9–84.2%) in the first three months of life and 68.1% (57.1–76.3%) through 6 months. These estimates were closely consistent across four independent studies from different countries (*I*^2^ = 0%) and concordant with randomised efficacy against severe RSV-associated lower respiratory tract illness (pooled 65%; 70%–82% for the licensed Pfizer vaccine). The safety profile was broadly reassuring: a small increase in preterm birth could not be excluded but was specific to the withdrawn GSK vaccine and not robustly significant for the licensed Pfizer vaccine; the apparent signal for hypertensive disorders of pregnancy was most consistent with residual confounding; and other obstetric and neonatal outcomes showed no increase. Reactogenicity was mild to moderate and transient.

### Effectiveness, efficacy, and waning of protection

The concordance between randomised efficacy and real-world effectiveness is a central result of this review. When matched by vaccine, outcome severity, and infant age, the two evidence streams essentially coincide: for the licensed Pfizer vaccine, efficacy against severe RSV-LRTI was 82.4% at 3 months and 70.0% at 6 months (18), closely mirroring the pooled real-world effectiveness against hospitalisation of 79.7% and 68.1% at the same time points (12–16). For this comparison, the licensed vaccine (Pfizer) estimate is the appropriate reference. The three-platform pooled figure of 65% is exploratory and should be read only as an approximate summary: its apparent statistical homogeneity (*I*^2^ = 0%) reflects the wide, overlapping confidence intervals of three trials that used different vaccines, severe disease definitions, and follow-up windows, rather than genuine comparability between them. Such agreement between controlled trials and real-world programmes conducted across four countries and diverse health systems is strong evidence that the protective effect is real and generalises to routine practice. It also addresses the gap identified by the 2024 Cochrane review (6), which was limited to randomised trials and called for well-designed observational evidence; the accrued real-world data now corroborate the trial findings rather than diverging from them.

Effectiveness was lower over the 0–6-month window than over the 0–3-month window, which is most plausibly explained by the waning of transplacentally acquired maternal antibody over the first months of life. This pattern is clinically reassuring because protection is greatest during the first months when the risk of severe RSV disease and hospitalisation peaks, while remaining substantial (~68%) through 6 months. It also has practical implications: the residual susceptibility later in infancy supports consideration of complementary infant passive immunisation (e.g., nirsevimab) for infants who enter their first RSV season beyond the period of strongest maternal protection, and it informs discussions of the optimal seasonal timing of maternal vaccination. Because the available estimates are cross-sectional at different age cut-offs rather than longitudinal, waning is inferred rather than directly measured within individuals, and dedicated age-stratified analyses would strengthen this inference. The dominant protective mechanism is transplacentally acquired maternal IgG, which is not replenished after birth (29); breast milk antibody is predominantly secretory IgA acting locally at mucosal surfaces and is not absorbed systemically (30), so breastfeeding is unlikely to prevent the waning of the systemic antibody that principally mediates protection against severe disease, although it may confer modest additional mucosal protection. Notably, the included studies did not report breastfeeding status or duration, so any contribution of breastfeeding, whether protective or as a marker of healthy vaccinee characteristics, remains unmeasured and represents a target for future research.

The immunogenicity reported by the randomised trials, with high transferred antibody at birth followed by decline over infancy, parallels the protection observed here and its attenuation through the first six months and is consistent with the effectiveness and efficacy findings.

### Safety: preterm birth

Preterm birth is the principal safety concern for maternal RSV vaccination. Across the randomised trials, the pooled risk ratio was 1.17 (95% CI 1.03–1.32; Mantel–Haenszel), a small but statistically significant increase; however, sensitivity analyses showed this signal to be neither robust nor generalisable. It was driven principally by the GSK RSVPreF3-Mat vaccine (GRACE (3); RR 1.37), which was consequently not advanced for maternal use: excluding this trial reduced the pooled estimate to a non-significant 1.10 (95% CI 0.95–1.27), and for the licensed Pfizer vaccine, the increase was small and not robustly significant (pivotal MATISSE 1.20, 0.98–1.46; three-trial Pfizer pool 1.21, 1.00–1.46, marginal and dependent on the exploratory phase-2b trial). Because all real-world evidence pertains to the Pfizer vaccine, the reassuring post-marketing findings and the not robustly significant Pfizer randomised estimate refer to the same product, whereas the clear signal derives from a different, withdrawn vaccine for which no real-world data exist. On balance, the evidence supports a small, product- and timing-dependent effect that is managed by current licensure rather than an established, generalisable harm.

Gestational age at vaccination is also relevant. The preterm-birth excess in the randomised programme arose in trials that vaccinated across a broad window (from 24 weeks), whereas the licensed Pfizer vaccine is administered only at 32–36 weeks, and the real-world studies using this restricted window showed no preterm increase, indicating that later vaccination mitigates the risk. Consistent with this, in the largest national safety cohort, the preterm point estimate was marginally higher in the subgroup vaccinated at 32 weeks or earlier (France, IRR 1.13, 95% CI 0.98–1.31) than overall (0.97). Later vaccination does not compromise infant protection: transplacental antibody transfer ratios were similar across maternal gestational age strata from 24 to 36 weeks Simões et al. (7), and effectiveness against infant hospitalisation in the 32–36-week programmes was high (79.7% at 0–3 months). The 32–36-week window therefore optimises the benefit–risk balance, preserving strong infant protection while minimising any preterm-birth signal.

The observational studies, pooled together, suggested that vaccination was associated with a lower risk of preterm birth (0.90, 95% CI 0.82–0.99), a biologically implausible finding that most likely reflects healthy vaccinee confounding, whereby vaccinated individuals are healthier and at a lower baseline risk (31, 32). The randomised (unconfounded) evidence therefore remains the more reliable guide to the causal effect. The effectiveness estimates are not vulnerable in the same way: the test-negative studies condition on infants presenting with acute respiratory illness, largely removing differential healthcare-seeking, and the population-based Scottish study is similarly insulated by whole-population linkage and matching; the resulting estimates closely matched the unconfounded randomised efficacy, whereas the observational protective preterm association is biologically implausible and contradicted by the randomised data. The effectiveness findings are therefore interpreted as reliable, even though the observational preterm signal is not. The same pattern appears, in the opposite direction, in the hypertensive disorders analysis, where confounding instead suggested harm. Although preterm birth, low birth weight, and stillbirth can themselves arise from maternal infection or other complications, the outcomes examined here were vaccine-safety endpoints, and maternal RSV vaccination does not cause infection. The single clear signal (preterm birth) was product-specific rather than infection-mediated, and the observational associations reflected confounding rather than a causal infective or inflammatory pathway.

Our preterm-birth findings are closely concordant with a recent preterm-focused meta-analysis (Kuitunen et al. (33)), which reported increased odds in randomised trials (OR 1.17, 95% CI 1.02–1.34) that lost statistical significance when restricted to the market-approved Pfizer vaccine (OR 1.21, 95% CI 0.98–1.49), with no association in observational data (OR 0.93). Our randomised estimate (Mantel–Haenszel RR 1.17) reproduces their pooled odds ratio (1.17) exactly, providing independent corroboration. That rapid review concluded that vaccination ‘seems to be associated with increased odds of preterm birth’, a framing that, taken in isolation, may overstate the clinical concern. By attributing the significant randomised signal specifically to the withdrawn GSK vaccine, demonstrating its mitigation by the licensed 32–36-week dosing window, incorporating a substantially larger body of real-world safety data, and identifying the observational estimate as a product of healthy vaccinee confounding, our analysis indicates that no robustly significant, generalisable increase in preterm birth is established for the licensed Pfizer vaccine as currently administered, offering a more clinically actionable interpretation of the same underlying data. Recent randomised-only syntheses have reached divergent conclusions on this outcome: whereas Kuitunen et al. (33) reported a significant increase, a contemporaneous meta-analysis (Lopes et al. (34)) obtained a similar point estimate (RR 1.15) that was non-significant (95% CI 0.95–1.38), and a further immunogenicity-and-safety meta-analysis (Saad et al. (35)) likewise found no significant difference (OR 1.09, 95% CI 0.87–1.37); both concluded that no preterm signal was present. This discrepancy illustrates that the effect is small and its statistical significance is sensitive to which trials are pooled; our product- and timing-resolved analysis reconciles these positions by localising the signal to the withdrawn GSK vaccine and to broader vaccination windows, and by showing it to be not robustly significant for the licensed Pfizer vaccine administered at 32–36 weeks.

### Safety: hypertensive disorders of pregnancy and other outcomes

The apparent association between maternal RSV vaccination and hypertensive disorders of pregnancy is best explained by residual confounding rather than a causal effect. Pooling all observational estimates yielded moderate heterogeneity (*I*^2^ = 25%), which was resolved almost entirely by stratifying, in a post-hoc exploratory analysis, on the degree of confounding control: studies with limited adjustment showed a modestly elevated but homogeneous estimate (1.13; *I*^2^ = 0%), whereas thoroughly adjusted studies converged on the null (1.01; *I^2^* = 0%), as did both randomised trials reporting this outcome (GRACE and MORISOT). This attenuation towards the null as adjustment improves is characteristic of confounding (31). Two additional observations reinforce this interpretation: the estimate from the single-centre study was strongly model-dependent (the standard adjusted odds ratio used in our pooled analysis was non-significant, 1.10, 95% CI 0.90–1.35, rising to 1.43 only in a time-dependent Cox model, whose handling of immortal time is prone to overestimation), and the limited-adjustment surveillance analysis generated a scatter of borderline associations across several unrelated outcomes that were significant only against the concurrent comparator and unreplicated elsewhere, a pattern more consistent with incomplete confounding control than with a coherent set of causal effects. A specific unadjusted confounder, a higher prevalence of assisted reproduction among the vaccinated, was documented in one such study. A small causal effect cannot be entirely excluded, and continued surveillance is warranted, but the best-adjusted observational and the randomised evidence do not support a true increase. Other obstetric and neonatal outcomes, including small-for-gestational-age birth, low birth weight, stillbirth, and congenital anomalies, showed no increase, and no new safety signal emerged on complete-panel review.

### Comparison with previous systematic reviews

Our findings both corroborate and extend recent syntheses. Earlier randomised trial meta-analyses, including the 2024 Cochrane review (6), the updated 2025 Cochrane review (36), and other recent analyses (33–35, 37, 38), consistently found maternal RSVpreF vaccination to be efficacious with a reassuring perinatal safety profile; notably, the 2025 Cochrane review graded the efficacy of maternal vaccination against medically attended (VE 54%) and severe (VE 74%) infant RSV-LRTI and against hospitalisation (VE 54%) as high-certainty evidence, closely matching our own efficacy estimates and GRADE ratings. The present review differs in several important respects. First, it integrates the now-substantial body of real-world evidence, comprising fifteen observational effectiveness and safety studies of the licensed Pfizer vaccine, alongside the randomised trials. This directly fills a gap that randomised-only syntheses could not: as recently as a living systematic review with searches to March 2025 (39), no real-world effectiveness data for the maternal RSV vaccine were yet available. Our pooled effectiveness against infant RSV hospitalisation (approximately 80% within 5 months) is highly concordant with the only other real-world synthesis to report this outcome (79.1%; Oliva and Oliveira (40)) and with randomised efficacy, providing triangulated evidence that the protective effect is real and applies in routine practice. Second, whereas randomised-only pooling characterises the preterm birth question in aggregate, our sensitivity analyses localised the statistically significant signal to the withdrawn GSK vaccine, showed it to be not robustly significant and gestational window-mitigated for the licensed Pfizer product, and revealed that the apparent protective association in observational data reflects healthy vaccinee confounding, distinctions with direct clinical and regulatory relevance. Third, our search was more recent and therefore captured the effectiveness and post-marketing safety data that have accrued across multiple national programmes in 2024–2026. In these respects, the present review provides an evidence base that is more complete and more directly anchored in real-world data than existing randomised-only analyses.

### Geographic representation and generalisability

A further consideration is geographic representation. The randomised efficacy evidence was multinational and included some sub-Saharan African representation: the pivotal MATISSE trial enrolled participants across 18 countries on several continents, including South Africa; the Novavax PREPARE trial included South African sites; and the MORISOT trial was conducted with pregnant women in South Africa, including those living with HIV. This representation is nonetheless limited, being largely confined to a single upper-middle-income country rather than the low-income settings where the RSV burden is greatest. The real-world effectiveness and post-marketing safety evidence, however, derived entirely from high-income settings (the United Kingdom, Argentina, France, and the United States), with no effectiveness or large-scale safety data yet available from low- and middle-income countries (LMICs) or from Asia. This gap matters because the global burden of RSV is overwhelmingly concentrated in LMICs: of an estimated 101,000 RSV-attributable deaths in children younger than 5 years each year, more than 97% occur in LMICs, as do roughly 95% of RSV lower respiratory tract infection episodes (1). Whether the effectiveness, optimal seasonal timing, and perinatal safety profile observed in high-income programmes generalise to these settings has not yet been confirmed with real-world data and could plausibly differ owing to near-equatorial year-round RSV transmission, higher background rates of preterm birth and low birth weight, a higher prevalence of maternal HIV and other comorbidities, and more limited access to hospital care and to infant nirsevimab. Because a single antenatal dose can protect the newborn through the highest-risk early months without requiring infant contact with the health system, maternal vaccination is especially attractive for LMICs; generating real-world effectiveness and safety evidence from African and Asian programmes is therefore both a research and an implementation priority.

### Strengths and limitations

Strengths of this review include the integration and separate analysis of randomised and observational evidence, which enabled the efficacy–effectiveness comparison and the confounding-aware interpretation of the safety signals; rigorous, evidence-based handling of overlapping populations, verified from study periods and named participating sites rather than assumed; a complete outcome-panel extraction that confirmed no safety signal was overlooked; pre-specified sensitivity analyses that established the robustness of the effectiveness estimates and the fragility of the preterm signal; and a GRADE certainty rating of each outcome. Notably, all real-world evidence concerned the licensed Pfizer vaccine, so the effectiveness and post-marketing safety findings apply directly to the product in clinical use.

Several limitations should be acknowledged. Screening, data extraction, and risk-of-bias assessment were performed by a single reviewer with large language model-assisted verification rather than by two independent human reviewers; although this person–machine approach was pre-specified in the registered protocol and all decisions were checked against the primary sources, single-reviewer screening remains less robust than that of two independent human reviewers and is acknowledged as a limitation. Because no single outcome included ten or more studies, publication bias could not be formally assessed, and small-study effects cannot be excluded. The randomised efficacy pool combined three different vaccine platforms that also differed in their definitions of severe disease and their follow-up windows, so the three trials were not directly comparable; the pooled efficacy estimate is therefore best regarded as an approximate cross-platform summary, with the licensed Pfizer vaccine taken as the primary reference. One included safety dataset was available only as a conference abstract and could not be fully appraised. The observational studies, despite adjustment, remain susceptible to residual confounding, as the preterm and hypertensive disorder analyses illustrate. Because these studies reported different adjusted ratio measures (odds ratios, risk ratios, incidence rate ratios, and adjusted risk reductions), they were pooled as approximately equivalent measures, an approximation that is least accurate for higher-incidence outcomes such as hypertensive disorders of pregnancy. Finally, most evidence derived from high-income settings, and data from low- and middle-income countries, where the RSV burden is greatest, and from special populations remain comparatively limited; in particular, no real-world effectiveness or post-marketing safety data were available from African or Asian programmes. In addition, a subset of Embase records (118 of 2,146) was retrievable only in PDF format and could be de-duplicated on title alone, so a small number of duplicate or near-duplicate records may have persisted or been imperfectly handled during screening. The included studies did not report maternal RSV or other respiratory virus infection episodes during pregnancy; because maternal immunisation is intended to protect the infant rather than to prevent maternal infection, maternal respiratory infection was not an assessed outcome, and its relationship to birth outcomes could not be examined.

### Implications and future directions

These findings support the continued use of maternal RSVpreF vaccination as an effective strategy to prevent severe infant RSV disease, administered within the licensed 32–36-week gestational window that mitigates the concern of preterm birth. The waning of protection through infancy underscores the complementary role of infant monoclonal antibody prophylaxis for infants inadequately covered by maternal immunisation and supports attention to seasonal timing. Continued pharmacovigilance for preterm birth and hypertensive disorders is warranted, ideally through large, well-adjusted cohorts with active comparators capable of minimising healthy vaccinee bias and through analyses that distinguish product- and timing-specific effects. Further real-world effectiveness and safety data from low- and middle-income settings, particularly from sub-Saharan Africa and Asia, and from special populations, together with longer-term follow-up and age-stratified analyses of the duration of protection, will refine the evidence base and inform global implementation.

## Conclusion

Maternal RSVpreF vaccination substantially and reproducibly protects infants against RSV-associated lower respiratory tract infection and hospitalisation. By integrating randomised efficacy with real-world effectiveness from national immunisation programmes, this review shows that the two evidence streams closely coincide: effectiveness against hospitalisation of approximately 80% in the first three months of life, which is concordant with trial efficacy against severe disease, confirming that the benefit observed in trials carries over to routine practice. The principal safety consideration, a small increase in preterm birth, was chiefly attributable to the withdrawn GSK vaccine and was not robustly significant for the licensed Pfizer vaccine administered within its approved 32-36-week window; the apparent protective association in observational data reflects healthy vaccinee confounding rather than a true benefit. For the maternal RSV vaccine in current clinical use, the balance of evidence favours a meaningful infant benefit with a reassuring, well-characterised safety profile, supporting its continued programmatic use while longer-term post-marketing surveillance is accrued.

## Data Availability

The original contributions presented in the study are included in the article/Supplementary material, further inquiries can be directed to the corresponding author.
