# Supplementary material for: Efficacy and safety of maternal RSV vaccination against infant lower respiratory tract infections: a systematic review and meta-analysis
Source: Front Public Health. 2026 Sep 18;14:1935421. doi: 10.3389/fpubh.2026.1935421 (PMC13630920; doi:10.3389/fpubh.2026.1935421)
Supplement: Supplementary file 1 [file Data_Sheet_1.docx]

**Supplementary Material**

*Maternal RSV vaccination for prevention of infant lower respiratory tract infection: a systematic review and meta-analysis.*

# Supplementary Material S1. Search strategies

**Full electronic search strategies for each database, with dates.**

| Search Strategy (PRISMA-S documentation) |  |
| --- | --- |
| Concepts | Concept A = RSV • Concept B = pregnancy/maternal • Concept C = vaccine/immunization. Final = A AND B AND C. |
| Limits | NO study-design filter (protocol includes cohort/non-randomized). NO date limit (inception to search date). English limit optional — better to filter at screening. Record date run + hit count. |
| ① PubMed / MEDLINE — paste each set; run, then combine #4 AND #8 AND #13 |  |
| #1 | "Respiratory Syncytial Viruses"[Mesh] OR "Respiratory Syncytial Virus Infections"[Mesh] OR "Respiratory Syncytial Virus Vaccines"[Mesh] |
| #2 | RSV[tiab] OR "respiratory syncytial virus"[tiab] OR "respiratory syncytial viruses"[tiab] |
| #3 | (none — go to combine) |
| #4 (A) | #1 OR #2 |
| #5 | "Pregnancy"[Mesh] OR "Pregnant Women"[Mesh] OR "Prenatal Care"[Mesh] OR "Pregnancy Trimester, Third"[Mesh] |
| #6 | matern*[tiab] OR pregnan*[tiab] OR prenatal[tiab] OR antenatal[tiab] OR gestation*[tiab] OR "in utero"[tiab] OR transplacental*[tiab] OR mother*[tiab] |
| #7 | (none — go to combine) |
| #8 (B) | #5 OR #6 |
| #9 | "Vaccination"[Mesh] OR "Vaccines"[Mesh] OR "Immunization"[Mesh] OR "Immunization, Passive"[Mesh] |
| #10 | vaccin*[tiab] OR immuni*[tiab] OR RSVpreF[tiab] OR RSVPreF3[tiab] OR "prefusion F"[tiab] OR "pre-fusion F"[tiab] OR nanoparticle[tiab] OR Abrysvo[tiab] OR Arexvy[tiab] OR "PFP-2"[tiab] OR ResVax[tiab] |
| #11 | (none — go to combine) |
| #13 (C) | #9 OR #10 |
| #FINAL | #4 AND #8 AND #13 |
| One-box version | ("Respiratory Syncytial Viruses"[Mesh] OR "Respiratory Syncytial Virus Infections"[Mesh] OR "Respiratory Syncytial Virus Vaccines"[Mesh] OR RSV[tiab] OR "respiratory syncytial virus"[tiab] OR "respiratory syncytial viruses"[tiab]) AND ("Pregnancy"[Mesh] OR "Pregnant Women"[Mesh] OR "Prenatal Care"[Mesh] OR "Pregnancy Trimester, Third"[Mesh] OR matern*[tiab] OR pregnan*[tiab] OR prenatal[tiab] OR antenatal[tiab] OR gestation*[tiab] OR "in utero"[tiab] OR transplacental*[tiab] OR mother*[tiab]) AND ("Vaccination"[Mesh] OR "Vaccines"[Mesh] OR "Immunization"[Mesh] OR "Immunization, Passive"[Mesh] OR vaccin*[tiab] OR immuni*[tiab] OR RSVpreF[tiab] OR RSVPreF3[tiab] OR "prefusion F"[tiab] OR "pre-fusion F"[tiab] OR nanoparticle[tiab] OR Abrysvo[tiab] OR Arexvy[tiab] OR "PFP-2"[tiab] OR ResVax[tiab]) |
| ② Cochrane CENTRAL (Cochrane Library → Advanced Search → Search Manager) |  |
| #1 | MeSH descriptor: [Respiratory Syncytial Viruses] explode all trees |
| #2 | MeSH descriptor: [Respiratory Syncytial Virus Infections] explode all trees |
| #3 | MeSH descriptor: [Respiratory Syncytial Virus Vaccines] explode all trees |
| #4 | (RSV or "respiratory syncytial virus" or "respiratory syncytial viruses"):ti,ab,kw |
| #5 | #1 OR #2 OR #3 OR #4 |
| #6 | MeSH descriptor: [Pregnancy] explode all trees |
| #7 | MeSH descriptor: [Pregnant Women] explode all trees |
| #8 | (matern* or pregnan* or prenatal or antenatal or gestation* or transplacental* or "in utero" or mother*):ti,ab,kw |
| #9 | #6 OR #7 OR #8 |
| #10 | MeSH descriptor: [Vaccination] explode all trees |
| #11 | MeSH descriptor: [Vaccines] explode all trees |
| #12 | MeSH descriptor: [Immunization] explode all trees |
| #13 | (vaccin* or immuni* or RSVpreF or RSVPreF3 or "prefusion F" or nanoparticle or Abrysvo or Arexvy or "PFP-2" or ResVax):ti,ab,kw |
| #14 | #10 OR #11 OR #12 OR #13 |
| #15 | #5 AND #9 AND #14 ← FINAL (then filter to 'Trials' / CENTRAL) |
| How to run + record |  |
|  | • PubMed: pubmed.ncbi.nlm.nih.gov → paste the One-box version → Search → note total count → 'Save' > 'Send to' > Citation manager (.nbib) → import to a reference/screening manager/Zotero. |
|  | • Cochrane CENTRAL: cochranelibrary.com → Advanced search → Search manager → enter lines #1–#15 → run #15 → restrict results to 'Trials' (CENTRAL) → Export (RIS). |
|  | • Record each database's hit count + date on the 'PRISMA Flow Counts' tab. Keep the exact strings here unchanged — this tab IS your published search appendix. |
|  | • Embase and Scopus full strategies are documented below (sections ③ and ④). All four databases were searched on 15 June 2026 (PubMed 1,232; Embase 2,146; Scopus 1,865; Cochrane CENTRAL 172). |
| ③ Embase (embase.com → Advanced Search) |  |
| Full query | ('respiratory syncytial virus'/exp OR rsv:ti,ab,kw OR 'respiratory syncytial virus':ti,ab,kw) AND ('pregnancy'/exp OR 'pregnant woman'/exp OR matern*:ti,ab,kw OR pregnan*:ti,ab,kw OR prenatal:ti,ab,kw OR antenatal:ti,ab,kw OR gestation*:ti,ab,kw OR transplacental*:ti,ab,kw OR mother*:ti,ab,kw) AND ('vaccination'/exp OR 'vaccine'/exp OR 'immunization'/exp OR vaccin*:ti,ab,kw OR immuni*:ti,ab,kw OR rsvpref:ti,ab,kw OR 'prefusion f':ti,ab,kw OR nanoparticle:ti,ab,kw OR abrysvo:ti,ab,kw OR arexvy:ti,ab,kw OR resvax:ti,ab,kw) |
| Run + record | Log in via institution at embase.com → Search → Advanced Search → paste the query → run → record result count + date (Embase n = 2,146; 15 June 2026). Embase indexes many conference abstracts: retain them here and exclude 'conference abstracts without sufficient data' later at screening (per protocol). Select all → Export → RIS (for a reference/screening manager, e.g. Zotero or EndNote) or CSV, including title, abstract and keywords → download. |
| ④ Scopus (scopus.com → Advanced document search) |  |
| Full query | TITLE-ABS-KEY("respiratory syncytial virus" OR rsv) AND TITLE-ABS-KEY(matern* OR pregnan* OR prenatal OR antenatal OR gestation* OR transplacental* OR mother*) AND TITLE-ABS-KEY(vaccin* OR immuni* OR rsvpref OR "prefusion f" OR nanoparticle OR abrysvo OR arexvy OR resvax) |
| Run + record | scopus.com → Advanced document search → paste the query → Search → record result count + date (Scopus n = 1,865; 15 June 2026) → Export → RIS or CSV, including title, abstract and keywords → import to reference/screening manager. |
| *Abbreviations: MeSH, Medical Subject Headings (PubMed/MEDLINE); Emtree, Embase subject-heading thesaurus; ti,ab,kw, title/abstract/keyword field search; exp, exploded subject-heading tree; AND/OR, Boolean operators; DOI, Digital Object Identifier; RIS, Research Information Systems citation-file format; CSV, comma-separated values; PRISMA-S, PRISMA search-reporting extension; RSVpreF/RSVPreF3, RSV prefusion F protein-based vaccines (Pfizer/GSK).* | |

# Supplementary Material S2. PRISMA 2020 flow counts and checklists

*The completed PRISMA 2020 (27-item) and PRISMA-S checklists are provided in the accompanying audit file. Record counts are summarised below.*

| PRISMA 2020 Flow — Record Counts |  |  |
| --- | --- | --- |
| Records identified — PubMed/MEDLINE | 1232 | PubMed/MEDLINE — run 14 June 2026 (n=1,232) |
| Records identified — Embase | 2146 | Embase — run 15 June 2026 (n=2,146) |
| Records identified — Cochrane CENTRAL | 172 | Cochrane CENTRAL — run 15 June 2026 (n=172) |
| Records identified — Scopus | 1865 | Scopus — run 15 June 2026 (n=1,865) |
| Records from other sources (reference lists, registries) | 0 |  |
| TOTAL records identified | 5415 |  |
| Duplicates and non-machine-readable records removed | 2,674 | 2,556 duplicates (DOI, then normalised title/year) + 118 records retrievable only as non-machine-readable PDF (de-duplicated on title only). 5,415 − 2,674 = 2,741. |
| Records screened (title/abstract) | 2741 | Unique records after de-duplication = 2,741 (includes the 118 non-machine-readable Embase PDF records, de-duplicated on title alone). |
| Records excluded at title/abstract | 2658 | Records excluded at title/abstract screening = 2,741 − 83 = 2,658. |
| Reports sought for full-text retrieval | 83 |  |
| Reports not retrieved | 0 |  |
| Reports assessed for eligibility (full-text) | 83 |  |
| Reports excluded at full-text (total) | 54 | Grouped by PRISMA category below; full operational reasons are listed in Supplementary Material S4. |
| — Wrong population | 4 | Non-pregnant populations. |
| — Wrong intervention/comparator | 6 | Nirsevimab, natural infection, or wrong comparator/co-intervention. |
| — No eligible outcome | 7 | Immunogenicity, uptake, or maternal infection only — no eligible infant/safety outcome. |
| — Wrong study design | 22 | Reviews, editorials, commentary or news; modelling/cost-effectiveness; and case series or VAERS reports without a comparator. |
| — Other / abstract only | 15 | Ongoing studies without results; duplicate or companion reports of included studies; and animal, descriptive, or non-English reports. |
| STUDIES INCLUDED in review | 24 | 24 studies (9 RCTs + 15 observational), reported in 29 articles incl. companions. 83 assessed - 54 excluded = 29 reports. |
| Studies included in meta-analysis | Effectiveness pool 4-6; Efficacy 3; Preterm RCT 7 / obs 5 primary (7 incl. VSD+Blauvelt sensitivity); HDP 4 |  |
| *Abbreviations: DOI, Digital Object Identifier; PRISMA, Preferred Reporting Items for Systematic Reviews and Meta-Analyses; RCT, randomised controlled trial; HDP, hypertensive disorders of pregnancy; VAERS, Vaccine Adverse Event Reporting System; VSD, Vaccine Safety Datalink.* | | |

# Supplementary Material S3. Characteristics of included studies

| Characteristics of included studies (24 studies: 9 randomised trials, 15 observational) |  |  |  |
| --- | --- | --- | --- |
| A) RANDOMISED CONTROLLED TRIALS (RoB 2) — 9 trials |  |  |  |
| Trial / family | **Index + companion reports** | **Vaccine (sponsor)** | **Note** |
| MATISSE | Kampmann 2023; Simões 2025; Madhi 2025; Shittu 2026; Otsuki 2024 | RSVpreF (Pfizer) | Efficacy from final analysis (Simões 2025) |
| GRACE / MAT-009 | Dieussaert 2024; Banooni 2026 | RSVPreF3-Mat (GSK) | Stopped early (preterm signal) |
| Prepare | Madhi 2020 (+ Novavax abstracts) | RSV F nanoparticle (Novavax) | Phase 3 |
| Novavax phase 2 | Muñoz 2019 | RSV F nanoparticle (Novavax) | Safety/immunogenicity |
| Pfizer phase 2b | Simões 2022 | RSVpreF (Pfizer) | Safety/immunogenicity |
| GSK MAT-004 | Bebia 2023 | RSVPreF3 (GSK) | Phase 2 |
| GSK high-risk | Bebia 2026 | RSVPreF3-Mat (GSK) | High-risk women; stopped early |
| MORISOT | Myer 2025 | RSVpreF (Pfizer) | HIV+ women |
| Wyeth PFP-2 | Muñoz 2003 | PFP-2 (Wyeth) | Small RCT |
| B) OBSERVATIONAL — real-world effectiveness & safety (Newcastle-Ottawa) |  |  |  |
| Study | **Country / design** | **Type** | **Overlap cluster** |
| BERNI (Pérez Marc 2025) | Argentina / test-negative CC | Effectiveness | Argentina cluster — primary study (encompasses Gentile and OBS-AR3) |
| Gentile 2025 | Argentina / test-negative CC | Effectiveness | Argentina cluster — sensitivity only |
| Razzini 2025 (RIMA) | Argentina / cohort+TNCC | Effectiveness | Argentina cluster — sensitivity only |
| Williams & Marlow 2025 | UK / test-negative CC (2024-25) | Effectiveness | UK/Scotland cluster (2024-25) — sensitivity only (overlaps McLachlan) |
| O'Hagan / PERUKI-BronchStart 2026 | UK / test-negative CC (2025-26) | Effectiveness | UK season 2 (2025-26) — independent |
| McLachlan 2025 (Scotland) | Scotland / nested CC + cohort | Effectiveness | Scotland season 1 — primary study (preferred over Williams) |
| Rick 2025 (CASSATT) | USA / test-negative CC | Effectiveness | Independent |
| Son 2024 | USA NYC / cohort | Safety | Independent (single New York City health system) |
| Solsman 2026 (Utah) | USA Utah / cohort | Safety | Independent (statewide vital records) |
| Gabet 2026 (France) | France / national matched cohort | Safety | Independent (SNDS national database) |
| Kitano 2026 (TriNetX) | USA / TriNetX cohort | Safety | TriNetX cluster — Kitano retained; Hsieh excluded |
| Michnick 2025 (Sentinel) | USA / 5 health plans | Safety | Sentinel cluster — Michnick retained |
| VSD (conference abstract) | USA / VSD matched cohort | Safety | Overlaps Sentinel; sensitivity only |
| Blauvelt 2025 | USA / cohort | Safety (uptake-focused) | Low quality (uptake-focused) — sensitivity only |
| Razai 2025 (South London) | UK / cross-sectional | Safety (uptake-focused) | Low quality (uptake-focused) — sensitivity only |
| *Abbreviations: RCT, randomised controlled trial; RoB 2, Cochrane Risk of Bias 2 tool; NOS, Newcastle-Ottawa Scale; TNCC, test-negative case-control; CC, case-control; RSVpreF/RSVPreF3, RSV prefusion F protein-based vaccines (Pfizer/GSK); HIV, human immunodeficiency virus; SNDS, Systeme National des Donnees de Sante (French national health database); TriNetX, US federated electronic-health-record research network; VSD, Vaccine Safety Datalink.* | | | |

# Supplementary Material S4. Full-text records excluded, with reasons

| Exclusion reason | n |
| --- | --- |
| Not a primary study (review/editorial/commentary/news) | 16 |
| Not a primary study (modelling / cost-effectiveness) | 4 |
| Wrong outcome (immunogenicity / uptake / maternal infection) | 7 |
| Ongoing / no results yet | 6 |
| Duplicate / companion of an included study | 4 |
| Wrong population (non-pregnant) | 4 |
| Wrong intervention (nirsevimab / natural infection) | 3 |
| Wrong comparator / co-intervention | 3 |
| Excluded design (VAERS / case series, no comparator) | 2 |
| Other (animal, descriptive, non-English) | 5 |
| TOTAL full-text exclusions | 54 |
| *Abbreviations: VAERS, Vaccine Adverse Event Reporting System; RSV, respiratory syncytial virus.* | |

# Supplementary Material S5. Risk-of-bias assessments (RoB 2 for RCTs; Newcastle-Ottawa for observational)

**Per-study risk of bias. Full per-item justifications are in the workbook 'Risk of Bias' tab.**

| Study | Assessment / rating | Notes |
| --- | --- | --- |
| MATISSE (Kampmann 2023 / Madhi 2025) | Overall: Low | RoB 2 (per outcome). Phase 3, 1:1 double-blind placebo-controlled (NCT04424316); baseline balanced; independent data monitoring committee; RT-PCR-confirmed outcomes with blinded adjudication; pre-specified endpoints. Overall low risk of bias. |
| GRACE (Dieussaert 2024 / MAT-009) | Overall: Some concerns | RoB 2. Phase 3, 2:1 double-blind placebo-controlled; baseline balanced; independent adjudication committee; pre-specified endpoints. D3/Overall 'Some concerns' because enrolment was stopped early for the preterm-birth signal (5,328 of a planned ~10,000; ~53%), giving truncated, exploratory efficacy data (VE 65.5%, 95% credible interval 37.5-82.0, wide). The trial was stopped for potential harm, not benefit, so efficacy is not inflated; the resulting imprecision is carried into the GRADE assessment. |
| PREPARE (Madhi 2020 / Novavax RSV F) | Overall: Low | RoB 2. Phase 3, 2:1 observer-blind placebo-controlled; baseline balanced. The trial missed its primary efficacy endpoint (VE 39.4%, 97.52% CI -1.0 to 63.7) - a result, not a bias. D2: the primary analysis was per-protocol (PP), but the intention-to-treat (ITT) analysis gave the same ~39% point estimate (PP population 91.6%, ITT 98.9% of infants; exclusions modest and balanced), so the analysis choice did not distort the result - low. Objective outcome (oxygen saturation <95% plus RT-PCR) assessed by a separate blinded assessor. |
| Munoz 2019 (Novavax RSV F, phase 2) | Overall: Low | RoB 2. Phase 2, 1:1 (age-stratified) observer-blind saline-placebo (NCT02247726); descriptive safety/immunogenicity primary objective (not efficacy). Realised 28 vaccine / 22 placebo split due to slow site enrolment (logistics, not broken randomisation); Brighton Collaboration safety definitions; infants followed 1 year. N=50 only, so severely underpowered/imprecise: contributes safety reassurance and minimal meta-analysis weight; imprecision is handled in GRADE, not in risk of bias. |
| Simoes 2022 (Pfizer RSVpreF, phase 2b) | Overall: Low | RoB 2 (assessed per outcome). Phase 2b proof-of-concept, central 1:1:1:1:1, observer-blind placebo-controlled; baseline similar. Used here as a safety/immunogenicity study - low for those pre-specified outcomes. The efficacy signals (VE ~84.7% / 91.5%) were exploratory/post-hoc with no formal statistical testing, so they were not carried into the quantitative efficacy synthesis (narrative only), per the pre-specified rule. Interim n=406 women / 403 infants. |
| Bebia 2023 (GSK RSVPreF3, phase 2, MAT-004) | Overall: Low | RoB 2. Phase 2 observer-blind placebo-controlled, randomised 1:1:1 (60/120 ug / placebo; NCT04126213); safety/immunogenicity primary objective (no efficacy endpoint). N=213 vaccinated (~70/arm); 187 mothers / 176 infants completed; baseline similar (115 participants withdrew before randomisation and were excluded from the risk-of-bias assessment). The only notable vaccine-versus-placebo difference was higher reporting of hypertensive disorders of pregnancy (~10% vs lower), which feeds the cross-study HDP theme but rests on very small counts - hypothesis-generating/imprecise. |
| Bebia 2026 (GSK RSVPreF3-Mat phase 3, high-risk) | Overall: Low | RoB 2 (per outcome). Phase 3 double-blind placebo-controlled, 9 countries (NCT04980391 / EudraCT 2021-000994-96), high-risk pregnant women; safety/immunogenicity primary (no efficacy endpoint). Halted early on 25 Feb 2022 (sponsor-wide stop following the GRACE/MAT-009 signal): 186 enrolled / 169 analysed (vaccine 113, placebo 56); 94.1% completed; all followed for safety to study end. D3 = low: early termination reduced the sample size (imprecision, handled in GRADE) but safety outcomes were complete - differing from GRACE, whose efficacy follow-up was truncated. |
| MORISOT (Myers 2025, RSVpreF in HIV+) | Overall: Low | RoB 2. Phase 3 randomised 1:1 double-blind placebo-controlled (NCT06325657), pregnant people living with HIV on antiretroviral therapy, South Africa; safety/immunogenicity primary (no efficacy endpoint). N=343 (172 vaccine / 171 placebo); 96.2% completed; baseline balanced. The special HIV-positive / HIV-exposed-uninfected (HEU) population affects generalisability - indirectness (GRADE), not risk of bias. Non-live recombinant subunit vaccine, so safety was comparable (AEs/SAEs similar including HDP; no preterm difference); infant immunogenicity was somewhat lower than in HIV-unexposed infants (geometric mean ratio HEU:non-HIV ~0.86). |
| Munoz 2003 (Wyeth PFP-2, pilot) | Overall: Some concerns | RoB 2. Randomised 4:3 double-blind saline-placebo pilot of RSV PFP-2 (an early subunit vaccine, not the current RSVpreF/RSVPreF3); safety/immunogenicity objective (no efficacy endpoint). N=35 only (20 vaccine / 15 placebo); all 35 infants accounted for. D5/Overall 'Some concerns' because the trial pre-dates mandatory trial registration (~2005), so there is no registered protocol to verify against (selective reporting unverifiable). N=35 also gives severe imprecision (trivial weight), and PFP-2 is a different-generation vaccine (indirectness); both are handled in GRADE. |
| BERNI (Perez Marc 2025) | 9/9 (Good) | Case-control NOS. Cases = hospitalised lower-respiratory-tract disease with lab-confirmed RSV (PCR/immunofluorescence); controls = same case definition, PCR-confirmed RSV-negative, same hospital network. Adjusted via inverse-probability-of-treatment weighting (IPTW) plus multilevel logistic regression (gestational-age window, calendar/programme timing, hospital clustering) - 2 comparability stars. Exposure from the national vaccine registry, identical for cases and controls and independent of case status; exclusions applied equally. |
| Scotland / McLachlan - S1 (2024-25) | 9/9 (Good) | Case-control NOS. Cases = RSV-LRTI hospitalisation <=90 days, PCR-confirmed; national population-based (all Scottish infants, record linkage SLiPBD/SMR01). Controls matched 10:1 by birth week and gestational age, no RSV-positive test. Comparability 2 stars: matched on gestational age and birth week and additionally adjusted (conditional logistic regression) for maternal age, birthweight, ethnicity, parity, infant sex, SIMD deprivation and smoking. Exposure from linked vaccination records, identical for all; whole-population (no non-response). |
| Son 2024 (NYC cohort) | 8/9 (Good) | Cohort NOS. Selection-1 (representativeness) withheld: single health system (2 New York City hospitals, selected affluent population, median age 34.9) - generalisability limit. Selection-2 same-source unexposed; Selection-3 exposure from electronic health records; Selection-4 perinatal outcomes post-vaccination with immortal-time bias handled (time-dependent Cox model). Comparability 2 stars: multivariable and Cox adjustment (maternal age, parity, insurance, race/ethnicity, clinical confounders). Outcome 3 stars: record-based, follow-up through delivery/neonatal period, minimal loss. |
| Utah (statewide cohort 2025) | 9/9 (Good) | Cohort NOS. Selection-1 statewide (all eligible Utah deliveries, N=24,213) - truly representative (contrast Son). Selection-2 same-source unexposed; Selection-3 exposure from the statewide immunisation registry; Selection-4 preterm birth post-vaccination, followed to delivery. Comparability 2 stars: adjusted odds ratio with backward selection including maternal age, history of preterm birth, urban residence and education (vaccinated women were older/urban/college-educated, so confounding-by-indication was addressed). Outcome 3 stars: birth and fetal-death certificates (record-based). |
| France (Gabet 2025, EPI-PHARE) | 9/9 (Good) | Cohort NOS. Selection-1 national (all French vaccinated women, SNDS national healthcare database) - truly representative. Selection-2 unvaccinated matched 1:1 from the same database; Selection-3 exposure from the national database; Selection-4 time-to-event and delivery-within-1/3-week analyses handle immortal-time bias. Comparability 2 stars: matched 1:1 on 7 factors (gestational age at vaccination, maternal age, region, week of conception, history of preterm birth, influenza vaccination, multiple pregnancy). Outcome 3 stars: validated administrative coding. |
| Sentinel (Michnick 2025) | 8/9 (Good) | Cohort NOS. Selection 4 stars (multi-site US insured population; concurrent active comparator = other-vaccine recipients in the same database; claims-based exposure; pre-specified outcomes). Comparability 1 star (2nd withheld): adjusted only by stratifying on research partner, gestational age at vaccination and maternal age; other confounders were deliberately not adjusted (design intended for rapid signal detection). The authors state that HDP/PROM elevations 'could not be ruled out due to limited confounding control'. Outcome 3 stars (claims-based, follow-up to delivery). |
| Kitano / TriNetX (npj Vaccines 2026) | 8/9 (Good) | Cohort NOS. TriNetX multi-healthcare-organisation US EHR network; propensity-score (PS)-matched 11,265 pairs. Selection 4 stars. Comparability 2 stars: PS-matched on extensive covariates including the key HDP confounders (pre-existing and gestational diabetes, baseline hypertension/HDP, obesity BMI categories, history of preterm labour) - which is why its HDP estimate is 1.01 (null), reinforcing the residual-confounding interpretation. Outcome O3 (follow-up adequacy) withheld: TriNetX may incompletely capture care sought outside the network. |
| VSD (abstract 1D-06) | Not assessable (Abstract-only) | Conference abstract only - full NOS not assessable (methods detail such as the full confounder set, representativeness and follow-up completeness were not reported). What is reported: VSD matched-pair cohort, index-date matching by gestational week, time-varying exposure with censoring/rematching (handles immortal-time bias), log-binomial regression. Preterm RR 0.90 (0.80-1.00; 571/14,099 vs 637/14,099); small-for-gestational-age RR 1.02 (0.93-1.13; 800/11,920 vs 781/11,920). Use = supporting/sensitivity evidence only. |
| Williams / UK1 - S1 (2024-25) | 8/9 (Good) | Case-control NOS. Multicentre test-negative, 30 UK sites, first season 2024-25; 537 pairs (391 RSV-positive cases, 146 RSV-negative controls). Selection 4 stars: lab-confirmed RSV-positive cases, test-negative controls from the same sites. Comparability 2 stars: conditional logistic regression adjusted for site, calendar month, infant age, gestational age, deprivation (IMD), maternal ethnicity and breastfeeding, plus a pertussis negative-control analysis. Exposure from medical records, identical for both. Exposure-3 (non-response) withheld under stricter appraisal. |
| O'Hagan / UK2 - S2 (2025-26) | 8/9 (Good) | Case-control NOS. Same PERUKI/BronchStart team and design as UK1, season 2/steady-state; 37 sites, 658 infants (402 RSV-positive cases, 256 RSV-negative controls; nirsevimab recipients excluded). Selection 4 stars; Comparability 2 stars (conditional logistic regression adjusted for site, calendar month, gestational age at birth, socioeconomic status/IMD, infant age and breastfeeding, plus a pertussis negative control). Exposure from medical records, identical for both; Exposure-3 non-response withheld (prospectively recruited/consent-based). |
| CASSATT (Rick 2025) | 8/9 (Good) | Case-control NOS. Retrospective test-negative, single health system (2 hospitals, Western Pennsylvania), interim; 274 infants (83 RSV-positive cases, 191 RSV-negative controls); VE 67.6%. Selection-representativeness withheld (single-centre, small interim - consistent with Son). Comparability 2 stars: multivariable logistic regression with directed-acyclic-graph (DAG)-based covariate selection (race, area deprivation index, age) and test-negative (healthcare-seeking) controls. Exposure from EHR plus the Pennsylvania Immunization Registry (verified), identical for both; record-based. |
| Gentile 2025 (Argentina) | 8/9 (Good) | Case-control NOS. Multicentre prospective test-negative nested; 187 infants (91 RSV-positive cases, 96 RSV-negative controls); adjusted VE 73%. Selection 4 stars. Comparability 1 star (2nd withheld, stricter): the multivariable logistic model was significance-driven, retaining only 3 covariates (age <3 months, prematurity, chronic respiratory disease); the small n=187 forced dropping others, and it was not adjusted for calendar time/season or maternal factors - the thinnest adjustment of the case-control set. Exposure from vaccination records, identical for both. |
| Razzini 2025 (RIMA, Buenos Aires) | 9/9 (Good) | Case-control NOS (VE from a nested test-negative case-control within the RIMA surveillance cohort). RT-PCR-confirmed cases; test-negative controls from the same surveillance. Comparability 2 stars: adjusted for age, sex, gestational age, comorbidities, health-insurance status, home location, plus epidemic-timing/season adjustment (richer than Gentile). Exposure from the Federal Vaccination Registry, identical for both; registry-based (no non-response). VE 80.8% (<3 months) / 66.1% (<6 months). Overlaps BERNI and Gentile (Argentina cluster). |
| South London (Razai 2025, BMJ Open) | 6/9 (FAIR) | Cross-sectional cohort NOS. Selection-representativeness withheld (single-centre London, selected: vaccinated women older). Comparability 0/2: perinatal-outcome comparisons were crude/unadjusted (authors' words: 'unadjusted comparisons, with potential for residual confounding'); the multivariable model addressed uptake predictors, not outcomes (e.g., a higher caesarean rate confounded by age). Outcome 3 stars (record-based, through delivery, complete). 911 eligible, 173 (19%) vaccinated - Fair quality; used for sensitivity only. |
| Blauvelt 2025 (UCSF, JAMA Netw Open) | 8/9 (Good) | Cohort NOS. Selection-representativeness withheld (single academic centre, UCSF; selected: older, high private insurance). Comparability 2 stars awarded because the study adjusted for or excluded confounding factors: a nested case-control for preterm birth used multivariable adjustment (age, parity, insurance, diabetes, obesity, multiple gestation, IVF), so the crude 8.5% (vaccinated) vs 18.5% (unvaccinated) attenuated to an adjusted odds ratio of 1.03 (0.55-1.93), demonstrating that the crude 'benefit' was confounding (only ~78 preterm events). |
| *Abbreviations: RoB 2, Cochrane Risk of Bias 2 tool; NOS, Newcastle-Ottawa Scale; ITT, intention-to-treat; PP, per-protocol; DMC, data monitoring committee; IPTW, inverse-probability-of-treatment weighting; PS, propensity score; GA, gestational age; HDP, hypertensive disorders of pregnancy; PROM, premature rupture of membranes; SGA, small-for-gestational-age; LRTI/LRTD, lower respiratory tract infection/disease; (S)IMD, (Scottish) Index of Multiple Deprivation; DAG, directed acyclic graph; HEU, HIV-exposed uninfected; EHR, electronic health record; VE, vaccine effectiveness/efficacy; VSD, Vaccine Safety Datalink.* | | |

# Supplementary Material S6. Extracted outcome data

**Study, design, outcome, effect measure and estimate with 95% CI. Full extraction (events/denominators, sources) is in the workbook 'Outcome extraction' tab.**

| Study | Design | Stream | Outcome | Measure | Est | CI low | CI high |
| --- | --- | --- | --- | --- | --- | --- | --- |
| MATISSE | RCT | Efficacy | Severe MA-RSV-LRTI | VE% | 82.4 | 57.5 | 93.9 |
| MATISSE | RCT | Efficacy | Severe MA-RSV-LRTI | VE% | 70.0 | 50.6 | 82.5 |
| MATISSE | RCT | Efficacy | MA-RSV-LRTI | VE% | 57.6 | 31.3 | 74.6 |
| MATISSE | RCT | Safety | Preterm birth | RR | 1.20 | 0.98 | 1.46 |
| MATISSE | RCT | Safety | Low birth weight | RR | 1.17 | 0.95 | 1.44 |
| MATISSE | RCT | Safety | Neonatal/infant death | RR | 0.57 | 0.24 | 1.36 |
| MATISSE | RCT | Safety | Congenital abnormalities | RR | 0.85 | 0.7 | 1.04 |
| GRACE | RCT | Efficacy | Any MA-RSV-LRTD | VE% | 65.5 | 37.5 | 82.0 |
| GRACE | RCT | Efficacy | Severe MA-RSV-LRTD | VE% | 69.0 | 33.0 | 87.6 |
| GRACE | RCT | Efficacy | RSV hospitalisation | VE% | 50.1 | -3.6 | 75.8 |
| GRACE | RCT | Safety | Preterm birth | RR | 1.37 | 1.08 | 1.75 |
| GRACE | RCT | Safety | Neonatal death | RR | 2.16 | 0.62 | 7.56 |
| GRACE | RCT | Safety | Low birth weight | RR | 1 | 0.79 | 1.26 |
| GRACE | RCT | Safety | Stillbirth |  |  |  |  |
| Prepare | RCT | Efficacy | RSV MA-significant LRTI | VE% | 39.4 | 5.3 | 61.2 |
| Prepare | RCT | Efficacy | RSV LRTI hospitalisation | VE% | 44.4 | 19.6 | 61.5 |
| Prepare | RCT | Efficacy | RSV-LRTI with severe hypoxemia | VE% | 48.3 | -8.2 | 75.3 |
| Prepare | RCT | Safety | Preterm birth | RR | 0.95 | 0.75 | 1.21 |
| Prepare | RCT | Safety | Stillbirth / fetal death | RR | 0.87 | 0.38 | 1.97 |
| Prepare | RCT | Safety | Low birth weight |  |  |  |  |
| Prepare | RCT | Safety | Congenital abnormalities |  |  |  |  |
| Simões 2022 | RCT | Efficacy | Any MA-RSV-LRTI (exploratory) | VE% | 84.7 | 21.6 | 97.6 |
| Simões 2022 | RCT | Efficacy | Severe MA-RSV-LRTI (exploratory) | VE% | 91.5 | -5.6 | 99.8 |
| Simões 2022 | RCT | Safety | (Efficacy = exploratory/post-hoc, U.S. cohort) | - |  |  |  |
| Simões 2022 | RCT | Safety | Preterm birth / pregnancy outcomes [CORRECTED: 14/325 vs 1/78; was 5/327 vs 1/79] | RR | 3.36 | 0.45 | 25.17 |
| Muñoz 2019 | RCT | Safety | (No clinical efficacy endpoint) | - |  |  |  |
| Muñoz 2019 | RCT | Safety | Preterm birth / pregnancy outcomes [CORRECTED: 1/22 vaccine vs 0/28 placebo; zero placebo cell. Standard RR not estimable; value shown is Haldane–Anscombe continuity-corrected (display only). Mantel–Haenszel pool uses raw uncorrected counts.] | RR (cc) | 3.8 | 0.2 | 89 |
| Bebia 2023 | RCT | Safety | (No clinical efficacy endpoint) | - |  |  |  |
| Bebia 2023 | RCT | Safety | Preterm birth / AESI |  |  |  |  |
| Bebia 2026 | RCT | Safety | (No clinical efficacy endpoint) | - |  |  |  |
| Bebia 2026 | RCT | Safety | Preterm birth | RR | 0.92 | 0.5 | 1.69 |
| Bebia 2026 | RCT | Safety | Neonatal death |  |  |  |  |
| MORISOT | RCT | Safety | (No clinical efficacy endpoint) | - |  |  |  |
| MORISOT | RCT | Safety | Preterm birth | RR | 1.12 | 0.55 | 2.28 |
| MORISOT | RCT | Safety | Hypertensive disorders of pregnancy | RR | 0.93 | 0.47 | 1.81 |
| Muñoz 2003 | RCT | Safety | (No clinical efficacy endpoint) | - |  |  |  |
| Muñoz 2003 | RCT | Safety | Perinatal/neonatal outcomes | - |  |  |  |
| BERNI | Obs | Effectiveness | RSV-LRTD hospitalisation | VE% | 71.3 | 53.3 | 82.3 |
| BERNI | Obs | Effectiveness | RSV-LRTD hospitalisation | VE% | 78.6 | 62.1 | 87.9 |
| BERNI | Obs | Effectiveness | Severe RSV-LRTD hospitalisation | VE% | 76.9 | 45.0 | 90.3 |
| OBS-UK1 | Obs | Effectiveness | RSV hospitalisation | VE% | 58 | 28 | 75 |
| OBS-UK1 | Obs | Effectiveness | RSV hospitalisation | VE% | 72 | 48 | 85 |
| OBS-UK2 | Obs | Effectiveness | RSV hospitalisation | VE% | 62 | 38 | 76 |
| OBS-UK2 | Obs | Effectiveness | RSV hospitalisation | VE% | 78 | 57 | 89 |
| OBS-SCOT | Obs | Effectiveness | RSV-LRTI hospitalisation | VE% | 82.2 | 75.1 | 87.3 |
| OBS-AR2 (Gentile) | Obs | Effectiveness | RSV hospitalisation | VE% | 78.7 | 51.4 | 90.7 |
| OBS-AR3 (Razzini) | Obs | Effectiveness | RSV-ALRTI hospitalisation | VE% | 80.8 | 62.8 | 90.5 |
| OBS-AR3 (Razzini) | Obs | Effectiveness | RSV-ALRTI hospitalisation | VE% | 66.1 | 30.1 | 83.8 |
| OBS-US-PA (CASSATT) | Obs | Effectiveness | RSV ARI hospitalisation | VE% | 67.6 | 33.2 | 85.4 |
| OBS-US-PA (CASSATT) | Obs | Effectiveness | RSV-LRTD hospitalisation | VE% | 69.0 | 25.5 | 88.0 |
| OBS-US-PA (CASSATT) | Obs | Effectiveness | RSV ARI hospitalisation | VE% | 74.2 | 25.4 | 93.0 |
| Son | Obs | Safety | Preterm birth | aOR | 0.87 | 0.62 | 1.20 |
| Son | Obs | Safety | Hypertensive disorders of pregnancy (time-dependent Cox model; NOT used in pool) | HR | 1.43 | 1.16 | 1.77 |
| OBS-US-UT | Obs | Safety | Preterm birth | aOR | 0.80 | 0.67 | 0.95 |
| OBS-FR | Obs | Safety | Preterm birth | IRR | 0.97 | 0.89 | 1.06 |
| OBS-US-EHR3 (Sentinel) | Obs | Safety | Preterm birth | ARR | 0.79 | 0.65 | 0.98 |
| OBS-US-VSD | Obs | Safety | Preterm birth | RR | 0.90 | 0.80 | 1.00 |
| OBS-SCOT | Obs | Effectiveness | RSV-LRTI hosp — PRETERM (<37 wk) subgroup | VE% | 89.9 | 55.3 | 97.7 |
| OBS-SCOT | Obs | Effectiveness | RSV-LRTI hosp — TERM (≥37 wk) subgroup | VE% | 81.5 | 73.9 | 87.0 |
| OBS-AR3 (Razzini) | Obs | Effectiveness | RSV PICU admission | VE% | 87.2 | 52.6 | 97.0 |
| OBS-AR3 (Razzini) | Obs | Effectiveness | Extended hospital stay | VE% | 88.6 | 62.3 | 97.1 |
| OBS-US-PA (CASSATT) | Obs | Effectiveness | RSV ARI hosp — TERM subgroup | VE% | 65.3 | 21.1 | 85.9 |
| OBS-US-PA (CASSATT) | Obs | Effectiveness | RSV ARI hosp — PRETERM subgroup | VE% | 63.5 | -101.7 | 95.4 |
| OBS-US-UT | Obs | Safety | NICU stay >24 h | aOR | 0.77 | 0.65 | 0.91 |
| OBS-FR | Obs | Safety | Stillbirth | IRR | 0.77 | 0.45 | 1.32 |
| OBS-FR | Obs | Safety | Small for gestational age | IRR | 1.01 | 0.96 | 1.07 |
| OBS-FR | Obs | Safety | Preeclampsia | IRR | 1.02 | 0.85 | 1.22 |
| OBS-US-VSD | Obs | Safety | Small for gestational age | RR | 1.02 | 0.93 | 1.13 |
| OBS-US-EHR3 (Sentinel) | Obs | Safety | Hypertensive disorders of pregnancy | ARR | 1.14 | 1.02 | 1.27 |
| OBS-US-EHR3 (Sentinel) | Obs | Safety | Premature rupture of membranes (PROM) | ARR | 1.09 | 0.97 | 1.22 |
| OBS-US-EHR3 (Sentinel) | Obs | Safety | Preterm PROM | ARR | 1.18 | 1.08 | 1.29 |
| OBS-US-EHR4 (Kitano/TriNetX) | Obs | Safety | Preterm birth | OR | 0.98 | 0.84 | 1.14 |
| OBS-US-EHR4 (Kitano/TriNetX) | Obs | Safety | Hypertensive disorders of pregnancy | OR | 1.01 | 0.96 | 1.08 |
| OBS-US-EHR1 (Hsieh/TriNetX) | Obs | Safety | (Maternal outcomes — TriNetX) | - |  |  |  |
| OBS-UK-LON (South London) | Obs | Safety | Preterm birth (<37 wk) | % |  |  |  |
| Blauvelt | Obs | Safety | Preterm birth | - |  |  |  |
| OBS-US-EHR4 (Kitano/TriNetX) | Obs | Safety | Gestational diabetes (GDM) | OR | 0.98 | 0.90 | 1.06 |
| OBS-US-EHR4 (Kitano/TriNetX) | Obs | Safety | Oligohydramnios | OR | 0.77 | 0.63 | 0.95 |
| OBS-US-EHR4 (Kitano/TriNetX) | Obs | Safety | Placental abruption | OR | 0.99 | 0.77 | 1.27 |
| OBS-US-EHR4 (Kitano/TriNetX) | Obs | Safety | Intrauterine growth restriction (IUGR) | OR | 0.94 | 0.86 | 1.03 |
| OBS-US-EHR4 (Kitano/TriNetX) | Obs | Safety | Intrauterine fetal death (IUFD) | OR | 0.68 | 0.38 | 1.22 |
| OBS-FR | Obs | Safety | Cesarean delivery | wIRR | 1.00 | 0.96 | 1.03 |
| OBS-FR | Obs | Safety | Postpartum hemorrhage | wIRR | 1.03 | 0.97 | 1.10 |
| OBS-FR | Obs | Safety | Major cardiovascular event | wIRR | 0.60 | 0.26 | 1.40 |
| OBS-US-EHR3 (Sentinel) | Obs | Safety | Stillbirth / LGA / SGA / LBW / preterm labor | - |  |  |  |
| Son | Obs | Safety | SGA / NICU / stillbirth / neonatal resp distress / jaundice | - |  |  |  |
| OBS-US-UT | Obs | Safety | HDP / low birth weight / SGA / fetal-infant death | - |  |  |  |
| Son | Obs | Safety | Small for gestational age | aOR | 1.16 | 0.89 | 1.50 |
| Son | Obs | Safety | Stillbirth | OR | 1.29 | 0.17 | 7.82 |
| Son | Obs | Safety | HDP (standard adjusted model; USED in limited-adjustment pool) | aOR | 1.10 | 0.90 | 1.35 |
| OBS-US-EHR3 (Sentinel) | Obs | Safety | Large for gestational age (LGA) | ARR | 1.33 | 1.03 | 1.67 |
| OBS-US-EHR3 (Sentinel) | Obs | Safety | Small for gestational age (SGA) | ARR | 1.11 | 0.81 | 1.48 |
| OBS-US-EHR3 (Sentinel) | Obs | Safety | Low birth weight | ARR | 0.93 | 0.56 | 1.45 |
| OBS-US-EHR3 (Sentinel) | Obs | Safety | Preterm labor without delivery | ARR | 0.82 | 0.65 | 1.01 |
| OBS-US-EHR3 (Sentinel) | Obs | Safety | Stillbirth | ARR | 0.25 | 0.08 | 0.57 |
| Abbreviations: RCT, randomised controlled trial; Obs, observational study; Est, point estimate; CI, confidence interval; VE, vaccine effectiveness/efficacy; RR, risk ratio; RR (cc), risk ratio with Haldane–Anscombe continuity correction; OR, odds ratio; aOR, adjusted odds ratio; HR, hazard ratio; IRR, incidence rate ratio; wIRR, weighted incidence rate ratio; ARR, adjusted rate ratio; RSV, respiratory syncytial virus; LRTI/LRTD, lower respiratory tract infection/disease; ARI, acute respiratory infection; MA, medically attended; HDP, hypertensive disorders of pregnancy; PROM, premature rupture of membranes; SGA/LGA, small-/large-for-gestational-age; LBW, low birth weight; IUGR, intrauterine growth restriction; IUFD, intrauterine fetal death; GDM, gestational diabetes mellitus; NICU/PICU, neonatal/paediatric intensive care unit; AESI, adverse event of special interest; TERM/PRETERM, infant gestational-age subgroups. Study codes (e.g. OBS-AR2, OBS-SCOT) are internal identifiers cross-referenced to Supplementary Material S3. | | | | | | | |

# Supplementary Material S7. Handling of overlapping (non-independent) populations

| Overlap resolution — non-independent study clusters (verified from source dates, hospital lists, data partners) |  |  |  |
| --- | --- | --- | --- |
| Cluster (stream) | In primary pool | Held out | Basis (verified) |
| UK effectiveness | Scotland / McLachlan (S1, 2024-25); O'Hagan / UK2 (S2, 2025-26) | Williams / UK1 -> sensitivity | Study periods: Scotland (12 Aug 2024-31 Mar 2025) and Williams (30 Sep 2024-20 Jan 2025) both Season 1 -> overlap (Williams includes Scottish sites); Scotland preferred (national linkage, n=27,565, NOS 9/9). O'Hagan (2 Sep 2025-31 Jan 2026) is Season 2 -> independent. |
| Argentina effectiveness | BERNI (Perez Marc) | Razzini; Gentile -> sensitivity | All 2024 season. Named hospital lists overlap in every pair: BERNI-Razzini = Elizalde + Ludovica; BERNI-Gentile = Vilela (Rosario, BERNI Site 11); Razzini-Gentile = Alejandro Posadas. BERNI chosen: national (12 hospitals across 7 provinces), largest (n=505), NOS 9/9, IPTW-adjusted. |
| US safety cohorts | Son; Sentinel; Utah; France; Kitano (TriNetX) | Hsieh -> dropped; VSD -> narrative only | Data sources verified distinct: Son (1 New York City health system), Sentinel (5 health plans), Utah (state vital records), France (SNDS national), Kitano (TriNetX). Hsieh uses the same TriNetX U.S. Collaborative as Kitano (2023-24) -> dropped; Kitano retained (full text, propensity-score matched). Sentinel shares Kaiser Permanente NW + HealthPartners with VSD -> not pooled together; VSD narrative/sensitivity only. |
| RCTs (efficacy & safety) | All 9 trials | - | Separate randomized trials with no inter-study population overlap. Pooled SEPARATELY from observational studies throughout. |
| US effectiveness | CASSATT (Rick 2025) | - (no overlap) | Independent - the sole US effectiveness study. Single health system, western Pennsylvania (2023-24 and 2024-25 seasons); test-negative, n=274, VE 67.6%, NOS 8/9. No overlap with UK or Argentina effectiveness studies (different countries). Distinct from the US safety cohorts by both data source and outcome (infant effectiveness vs maternal safety = different pools). Enters the effectiveness pool on its own. |
| Safety (sensitivity-tier) | Blauvelt 2025 (UCSF) | - (no overlap) | Independent data source: single academic centre, University of California San Francisco (UCSF), own electronic health record. Distinct from the other US safety cohorts (Son = NYC; Sentinel = 5 health plans; Utah = state; France = SNDS; Kitano = TriNetX). Theoretical minor overlap only if UCSF contributes to TriNetX, but the study used its own UCSF repository -> treated as distinct. Small and uptake-focused (NOS 8/9); used as sensitivity/supporting evidence -> no pool double-counting. |
| Safety (sensitivity-tier) | South London / Razai (City St George's) | - (narrative only) | Single London trust (City St George's, University of London), cross-sectional; measures uptake and perinatal outcomes (preterm/HDP), not infant RSV effectiveness. A London site could coincide with UK effectiveness sites (Williams/O'Hagan), but the different outcome (perinatal safety vs infant effectiveness) places it in a different pool, and it is used narratively/for sensitivity only (NOS 6/9 Fair, 0 comparability stars) -> not pooled -> no double-counting. |
| RCTs (report families) | All 9 trials (via Study Families tab) | Companion reports merged | Overlap among RCTs consists of multiple reports of the same trial, consolidated into families (MATISSE = Kampmann 2023 + Simoes 2025 + Madhi 2025 + Shittu 2026 + Otsuki 2024; GRACE = Dieussaert 2024 + Banooni 2026); each trial is counted once. Distinct trials (different sponsors/registrations) are independent. |
| *Abbreviations: NOS, Newcastle-Ottawa Scale; VE, vaccine effectiveness/efficacy; EHR, electronic health record; HDP, hypertensive disorders of pregnancy; SNDS, Systeme National des Donnees de Sante (French national health database); TriNetX, US federated electronic-health-record research network; VSD, Vaccine Safety Datalink; SLiPBD/SMR01, Scottish linked birth/hospital datasets.* | | | |

# Supplementary Material S8. Adverse events / reactogenicity narrative

| Adverse events / serious adverse events — narrative summary (NOT meta-analysed; described per study) |  |  |  |  |
| --- | --- | --- | --- | --- |
| Study | Maternal reactogenicity | Maternal AEs / SAEs | Infant / neonatal AEs / SAEs | Overall safety statement |
| MATISSE (Kampmann 2023; Simões 2025) | Injection-site reactions more common with vaccine; mostly mild–moderate | AEs and SAEs similar between groups; no new safety concerns | Comparable between groups (incl. low birth weight, hospitalisation) | No new safety concerns; favourable safety profile (final analysis) |
| GRACE (Dieussaert 2024; Banooni 2026) | Reactogenicity reported as acceptable and not the reason for termination; injection-site reactions more common with vaccine (consistent with other prefusion-F maternal vaccines), predominantly mild-moderate; no reactogen | Preterm-birth safety signal led to early termination; 'no other safety signal observed' | Neonatal death imbalance attributed to excess preterm births (13 vs 3) | Trial stopped early due to preterm-birth risk; otherwise no additional safety signal |
| Prepare (Madhi 2020) | Local injection-site reactions more common with vaccine (40.7% vs 9.9%) | Other AEs similar between groups; no systemic increase | No apparent negative impact on pregnancy/delivery/infant wellbeing | Well tolerated; no safety concerns identified |
| Muñoz 2019 (Novavax phase 2) | Injection-site reactions more frequent with vaccine (59% vs 4%), predominantly mild/transient injection-site pain; solicited systemic AEs similar proportions between groups. | No vaccine-related SAEs (small N=50) | No safety concerns in infants over 1-yr follow-up | Supports maternal immunisation safety; phase 2, small sample |
| Simões 2022 (Pfizer phase 2b) | Local reactions higher with vaccine (esp. aluminium-containing formulations); mostly mild–moderate | AEs similar between vaccine and placebo groups; no evident safety concerns | AE type/frequency consistent with background incidence in infants | No evident safety concerns; supported progression to phase 3 |
| Bebia 2023 (GSK MAT-004 phase 2) | Solicited local reactions more frequent with vaccine; solicited systemic and unsolicited AEs comparable between groups; well tolerated (predominantly mild/moderate). | Unsolicited AEs comparable; solicited higher with vaccine. HDP (gest. HTN/preeclampsia) more frequent in vaccine groups (not dose-dependent, within background rate) | No increase in preterm labour/birth or congenital anomalies vs placebo | Acceptable safety profile; possible HDP signal noted (see cross-study HDP theme) |
| Bebia 2026 (GSK high-risk, phase 3) | Well tolerated; reactogenicity reported | No vaccine-related SAEs | One neonatal death in each group; preterm birth balanced (18.2% vs 19.7%) | Acceptable safety profile in high-risk women; balanced preterm rates (study stopped early) |
| MORISOT (Myer 2025, HIV+ women) | Most reactogenicity mild/moderate | AEs and SAEs similar between groups; no safety concerns; HDP % reported between groups | No difference in preterm birth | Acceptable safety profile in pregnant women with HIV; no new safety concerns |
| Muñoz 2003 (Wyeth PFP-2) | Mild injection-site pain higher with vaccine (65% vs 13%, P=0.005); no systemic reactions or fever | No serious adverse events related to vaccine | All 35 infants born healthy; no differences in perinatal/neonatal outcomes or growth/development | Safe and well tolerated; very small early trial |
| *Abbreviations: AE, adverse event; SAE, serious adverse event; HDP, hypertensive disorders of pregnancy; HIV, human immunodeficiency virus; GBS, Guillain-Barre syndrome.* | | | | |

# Supplementary Material S9. GRADE Summary of Findings

| Summary of Findings (GRADE) — Maternal RSVpreF vaccination for prevention of infant RSV disease |  |  |  |  |
| --- | --- | --- | --- | --- |
| Outcome | Studies (design) | Pooled effect (95% CI) | Certainty (GRADE) | Reason for rating |
| RSV hospitalisation, infants 0-3 mo | 4 observational (case–control) | VE 79.7% (73.9-84.2) | MODERATE | Observational (start Low); upgraded +1 for large effect (RR ~0.20); consistent (I2=0%), precise, low risk of bias. |
| RSV hospitalisation, infants 0-6 mo | 3 observational (test-negative) | VE 68.1% (57.1-76.3) | MODERATE | Observational (start Low); upgraded +1 for large effect; consistent (I2=0%), precise. |
| Efficacy vs severe RSV-LRTI | 3 RCTs | VE 65.1% (49.1-76.1) | MODERATE | RCT (start High); downgraded -1 indirectness (three different vaccine platforms with differing severe-disease definitions and follow-up windows). MATISSE alone [licensed Pfizer] higher certainty. |
| Preterm birth | 9 RCTs / 7 pooled | RR 1.17 (1.03-1.32) | MODERATE | RCT (start High); downgraded -1 indirectness: signal is product-specific (driven by withdrawn GSK vaccine; not robustly significant for licensed Pfizer) and gestational-window-dependent (real-world 32-36 wk window shows n |
| Hypertensive disorders of pregnancy | 4 observational | 1.05 (0.99-1.12) | VERY LOW | Observational (start Low); -1 inconsistency (I²=25%; resolved only by adjustment stratum); -1 risk of bias (residual confounding; signal attenuates to null with better adjustment -> likely confounded). |
| Other obstetric/neonatal (SGA, LBW, stillbirth, congenital, etc.) | RCT + observational | All null / reassuring | MODERATE | No increase across outcomes; downgrade for imprecision on rare events (few events) offset by consistency. |
| Reactogenicity | 9 RCTs | Local reactions higher; systemic similar | -(narrative) | Not GRADE-rated (narrative); consistent mild-moderate transient local reactions, no reactogenicity SAEs. |
| *Abbreviations: GRADE, Grading of Recommendations Assessment, Development and Evaluation; RCT, randomised controlled trial; TN, test-negative; VE, vaccine effectiveness/efficacy; RR, risk ratio; CI, confidence interval; LRTI, lower respiratory tract infection; SGA, small-for-gestational-age; LBW, low birth weight; HDP, hypertensive disorders of pregnancy; RSVpreF, RSV prefusion F protein-based vaccine.* | | | | |

# Supplementary Material S10. Vaccine immunogenicity reported by the included randomised trials (narrative summary; not a pre-specified review outcome).

| Trial [ref] | Vaccine (platform) | Maternal neutralising-antibody response (fold-rise / GMR over pre-vaccination) | Transplacental (cord:maternal) transfer ratio | Infant antibody kinetics | Assay / notes |
| --- | --- | --- | --- | --- | --- |
| PREPARE (Madhi 2020) [4] | Novavax RSV F nanoparticle | Peak (day 14): palivizumab-competitive antibody 12.39-fold (95% CI 11.98–12.81); anti-F IgG 18.59-fold (17.84–19.36) over pre-vaccination | Palivizumab-competitive antibody 1.04 (1.02–1.06); anti-F IgG 1.17 (1.14–1.19) | Cord antibody far above placebo; infant half-life ~38–49 days (declined); maternal levels declined by day 180 post partum | Palivizumab-competitive antibody + anti-F IgG (ELISA); RSV-A/B microneutralisation |
| Simões 2022 [8] | Pfizer RSVpreF (phase 2b) | GMFR (delivery vs pre-vaccination): RSV A 11.0–15.1, RSV B 13.7–17.5 (across dose/adjuvant groups) | 1.41–2.10 (non-aluminium formulations 1.68–2.10; aluminium 1.41–1.67) | At birth, infant neutralising titres exceeded maternal levels (transfer ratio >1) | 50% RSV-A/B neutralisation |
| Bebia 2023 [9] | GSK RSVPreF3 (phase 2, healthy) | RSV-A 12.7 (60 µg) / 14.9 (120 µg); RSV-B 10.6 / 13.2 fold, 1 month post-vaccination | 1.62 (60 µg) and 1.90 (120 µg) | Highest at birth; declined through day 181 | Neutralising antibody |
| Bebia 2026 [10] | GSK RSVPreF3-Mat (phase 3, high-risk) | nAb GMR (delivery : pre-vaccination) RSV-A 8.87, RSV-B 8.21; anti-RSVPreF3 IgG 14.12-fold | 1.33 (RSVPreF3-specific IgG) | Successful placental transfer (ratio 1.33); infant kinetics over time not detailed | Neutralising Ab + IgG |

*Immunogenicity was not a pre-specified outcome of this review and was not formally synthesised; values are reported narratively from each trial and are not directly comparable across platforms because different neutralisation/antibody assays and reporting units were used. Representative trials are shown for each vaccine platform; other included randomised trials reported concordant immunogenicity (values available in the respective source publications). GMFR, geometric mean fold rise; GMR, geometric mean ratio; PCA, palivizumab-competitive antibody; nAb, neutralising antibody; IgG, immunoglobulin G.*

# Supplementary Material S11. Other obstetric and neonatal outcomes (forest plot).

Forest plot of other obstetric and neonatal outcomes in vaccinated versus unvaccinated or placebo pregnancies. Diamonds denote pooled estimates; squares denote single-study estimates; estimates cluster around the no-effect line. (The same outcomes are tabulated in main-text Table 4.) Diamonds, pooled estimates; squares, single-study estimates (log scale). CI, confidence interval; RCT, randomised controlled trial; obs, observational; VSD, Vaccine Safety Datalink; NICU, neonatal intensive care unit; PROM, prelabour rupture of membranes.
